# Computational Analysis of Excavatolide B–Human STING Interactions Implicates a Cys148–Adjacent Corridor with Within-Cavity Sub-Pose Diversity

**DOI:** 10.3390/ijms27052243

**Published:** 2026-02-27

**Authors:** Tien-Lin Chang, Hsiao-Yu Sun, Ping-Jyun Sung, Hsi-Wen Sun

**Affiliations:** 1 Institute of Biochemical and Biomedical Engineering, National Taipei University of Technology, Taipei 10608, Taiwan; shinya.chang@gmail.com; 2 Department of Life Science, National Taiwan University, Taipei 10617, Taiwan; suniverse2005@gmail.com; 3 National Museum of Marine Biology and Aquarium, Pingtung 94450, Taiwan; pjsung@nmmba.gov.tw; 4 Department of Marine Biotechnology and Resources, National Sun Yat-sen University, Kaohsiung 80424, Taiwan

**Keywords:** STING, briarane diterpenoid, colitis-associated colorectal cancer, computational docking, molecular dynamics, ADMET

## Abstract

Chronic, dysregulated inflammation contributes to colitis-associated colorectal cancer (CRC), and the cGAS–STING pathway represents a central but therapeutically challenging node because both insufficient and excessive STING activity can be pathogenic. Here, we integrate AlphaFold3 (AF3) receptor modeling, diffusion-based docking, and explicit-solvent molecular dynamics (MD) simulations to characterize how the marine briarane diterpenoid excavatolide B (ExcB) engages the human STING (hSTING) cyclic dinucleotide (CDN)-binding cleft. The structural integrity of the AF3 hSTING model was validated through both intrinsic confidence scores (pLDDT, PAE) and comparative benchmarking against experimental CTD structures (PDB: 4EF5, 6A05). Notably, the local geometries of key pocket-defining residues—including His157, Tyr167, and Thr263—remained consistent with established crystallographic data. Across three independent 100 ns MD replicas, ExcB exhibits a consistent spatial progression from an entrance-proximal pose at the solvent-accessible rim of the cleft (Site-2) to a more embedded, non-canonical corridor on the Cys148-adjacent side (Site-2′). Distance and contact analyses support a predominantly non-covalent within-cleft mechanism and do not indicate a persistent approach to the literature-reported covalent regime near Cys91. Residue-level profiling over the stabilized sampling window defines a reproducible corridor “contact signature” and reveals within-cavity sub-pose diversity rather than a single rigid bound pose. Mechanistically, competitive docking of the native agonist cGAMP to ExcB-conditioned receptor snapshots yields consistently less favorable docking outcomes in ExcB-conditioned conformations than docking to the native/open receptor; retaining ExcB coordinates does not further penalize cGAMP, supporting a receptor-reshaping (conformational conditioning) component rather than persistent static steric clash. Our findings characterize ExcB as a non-covalent modulator targeting a cryptic pocket within the STING CDN-binding cleft, establishing a structural basis for targeted mutagenesis and structure-activity relationship (SAR) studies.

## 1. Introduction

The stimulator of interferon genes (STING) adaptor is a central signaling node in innate immunity that links cytosolic DNA sensing to transcriptional programs driving type I interferon and inflammatory responses. Structural and mechanistic studies of the cGAS–STING axis have established that cyclic GMP–AMP (cGAMP) binding to the STING C-terminal domain (CTD) initiates conformational remodeling that enables downstream kinase recruitment and signaling propagation. Comprehensive overviews and structural syntheses of the pathway have clarified how ligand binding, conformational switching, and assembly states determine signaling output [1,2,3].

Beyond ligand activation, STING is modulated by multiple regulatory and inhibitory mechanisms. Covalent engagement of reactive cysteines has been demonstrated for non-nucleotide agonists and provides precedent that chemical reactivity and local microenvironments within the CTD can be exploited pharmacologically [4]. In parallel, redox regulation has been shown to suppress STING signaling through oxidative modification, with specific cysteine sites (including Cys148/Cys206) implicated by biochemical and proteomics evidence [5,6]. These observations collectively emphasize that STING is not a rigid receptor; rather, it is a conformationally plastic, chemically regulatable system in which transient accessibility and local geometry around the CDN-binding region can matter for function and inhibition.

A further layer of complexity is STING’s capacity to adopt higher-order assembly states. Polymer and oligomer architectures help rationalize how activation, hyperactivation, and inhibition might be tuned through changes in CTD geometry and inter-protomer packing [2,7]. From the perspective of structure-based inhibitor discovery, this plasticity implies that static experimental structures provide indispensable constraints yet may not fully report transient/cryptic connectivity within the CDN-binding cleft. Therefore, when proposing non-canonical microenvironments near the cleft rim or inner wall, it is essential to frame conclusions conservatively and to quantify dynamics using time-resolved analyses rather than over-interpreting any single structural snapshot.

Excavatolide B (ExcB) is a marine-derived briarane diterpenoid with reported anti-inflammatory and analgesic activities, including in vivo efficacy in inflammatory pain models [8]. Subsequent studies have also reported anti-inflammatory activity in disease contexts involving immune-cell and osteoclast-related processes [9]. More recently, ExcB has been discussed in the context of tissue repair and anti-inflammatory pharmacology, further motivating mechanistic investigation of its potential upstream immunomodulatory targets [10]. However, a direct structural mechanism linking ExcB to STING remains to be established experimentally, and it is not yet clear whether ExcB can access the STING CDN-binding cleft in a way that could plausibly interfere with ligand recognition events associated with STING activation.

In this work, we integrate receptor modeling, docking, and explicit-solvent molecular dynamics (MD) simulations to evaluate two tightly scoped, testable questions in silico: (i) whether ExcB can populate a reproducible microenvironment within the STING CDN-cleft region under explicit-solvent MD, and (ii) whether ExcB occupancy is accompanied by measurable changes in local inter-protomer “gate” geometry that could rationalize altered pocket accessibility. To bound model-dependence, we benchmark the starting CTD geometry against representative experimental CTD structures and report gate-region deviations as structural context. Throughout, we treat the proposed ExcB–STING interaction as a hypothesis-generating computational model, intended to prioritize experimentally testable residues and geometrical observables rather than to claim a definitive binding site absent direct structural or biochemical validation.

## 2. Results

We first evaluated whether ExcB can remain associated with the STING CDN-cleft region under explicit-solvent MD across three independent 100 ns replicas (R1–R3) initiated from identical coordinates and simulation settings but different randomized initial velocities. We then quantified two complementary readouts to support conservative, reproducible statements: (i) ligand confinement and recurrent residue-level contacts within the cleft region and (ii) time-resolved changes in a predefined set of inter-protomer “gate” metrics that report relative A/B chain geometry at positions bordering the CDN cleft. In parallel, we used mdpocket 4.0 (University of Paris, Paris, France) to estimate pocket/cavity descriptors over time (sampled every 2 ns) in order to describe cavity dynamics and replica-to-replica variability without assuming a single static pocket volume.

### 2.1. Docking Suggests an Entrance-Proximal Rim Pocket near Tyr167/Thr263 (Site-2)

**Figure 1:**
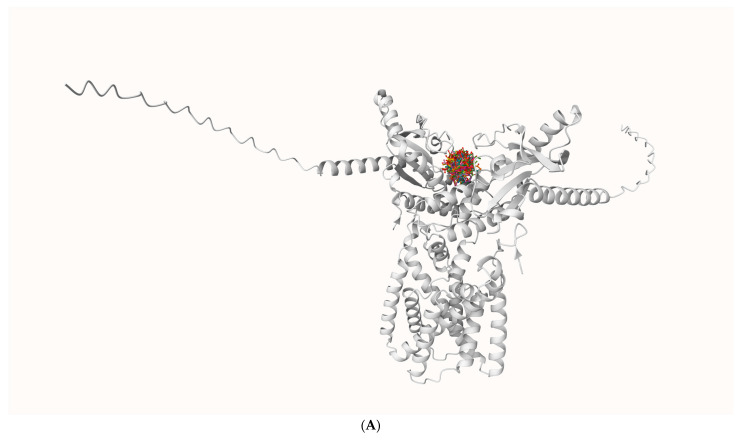

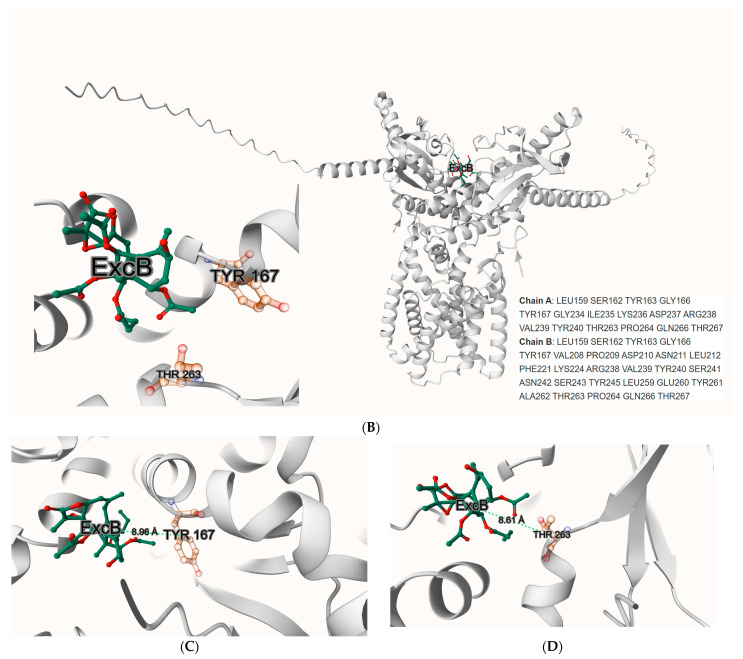
(**A**) DiffDock hotspot/clustering of the top 20 ExcB poses highlights a pocket (Site-2) on the rim of the CDN-binding cleft near Tyr167/Thr263. (**B**) AutoDock Vina-GPU re-docking/rescoring yields a consistent Site-2 pose (overlay shown with pocket surface). (**C**,**D**) Zooms centered on Tyr167 (**C**, **left**) and Thr263 (**D**, **right**) show ExcB remains outside H-bonding range; all annotated minimum heavy-atom distances (Å) exceed the 3.5 Å (0.35 nm) cutoff.

Using DiffDock v2.2.0 as an initial pose generator, we observed a recurrent cluster of ExcB poses at the solvent-accessible rim of the CDN-binding cleft, proximal to Tyr167 and Thr263 (Figure 1A). To obtain a consistent scoring reference, we re-docked and rescored representative poses with AutoDock Vina-GPU v2.1 (based on the AutoDock Vina 1.2.0 engine) (The Scripps Research Institute, La Jolla, CA, USA), which yielded favorable scores (typically in the −8 to −10 kcal·mol^−1^ range depending on pose and grid definition) and reproduced a similar rim-localized placement (Figure 1B). We refer to this entrance-proximal region as Site-2 (rim pocket) to distinguish it from the literature-reported covalent regime near Cys91 (Site-1) and from the deeper Cys148-adjacent corridor that becomes populated during MD (Site-2′). Docking alone does not establish a definitive binding site; we therefore use these poses only as MD starting hypotheses for evaluating whether ExcB can remain associated with the cleft region and whether its occupancy is accompanied by reproducible, quantifiable geometric changes.

### 2.2. Visual Separation from Tyr167/Thr263

Close-up views centered on Tyr167 and Thr263 confirm that ExcB sits in the cleft rim yet remains beyond H-bonding distance from both residues (Figure 1C,D) As annotated, all minimum heavy-atom distances exceed the standard H-bond cutoff of about 3.5 Å. This visual check anticipates the subsequent MD outcome: no stable hydrogen bonds are sustained with Tyr167 or Thr263. Instead, the Site-2 pose is stabilized by a distinct persistent polar anchor involving His157.

The binding affinity at this site is further corroborated by GPU-accelerated AutoDock Vina simulations, which predicted values ranging from ΔG = −8.0 to −10.3 kcal·mol^−1^, demonstrating the robustness and consistency of these interactions. These observations highlight the accessibility of the CDN-binding rim as a potential site for allosteric modulation. The spatial juxtaposition with residues implicated in cGAMP recognition, such as Tyr167 and Thr263, reinforces the relevance of this site in modulating (rather than mimicking) the conformational mechanics of STING activation.

While the established binding site involving residues Cys148 and Tyr240 (Site-1) is known to govern STING oligomerization and downstream signaling, the identification of the Tyr167/Thr263 pocket (Site-2) presents a fresh perspective. By focusing on this non-canonical rim pocket, our study provides new insights into how allosteric occupancy may bias cleft geometry and penalize CDN accommodation without direct competition. This opens up possibilities for the development of targeted therapeutic agents designed to fine-tune STING activity and its associated pathways.

### 2.3. Quantitative Gate-Geometry Changes Across Replicas

Building on the qualitative observations in Section 2.2, we quantified gate-region metrics and cavity descriptors across the three replicas to evaluate robustness and replica spread; detailed simulation and analysis procedures are described in Methods.

The AlphaFold3 (AF3, Google DeepMind, London, UK) hSTING CTD dimer was generated without a template and shows robust local and interface confidence of global pLDDT ≈ 77 with particularly high local accuracy at key gate residues (His157 ≈ 90; Tyr167 ≈ 89; Thr263 ≈ 88), ipTM = 0.79, pTM = 0.80, and an inter-protomer PAE median ≈ 9.7 Å. We relaxed the model (short minimization + restrained equilibration) and observed no change around the gate/pocket. Full per-residue pLDDT and the PAE map are provided in the SI (Figure S1A,B).

To validate the predicted topology, we benchmarked the AF3 model against experimentally solved hSTING CTD structures (PDB: 4EF5 and 6A05) using Cα superposition. The model shows high global agreement with PDB 4EF5 (global Cα RMSD = 1.03 Å over 177 common Cα atoms; chain mapping A → A), supporting the accuracy of the predicted fold. In contrast, superposition against PDB 6A05 yields a larger deviation (global RMSD = 5.31 Å over 364 common Cα atoms; chain mapping A → A and B → B), reflecting the structural heterogeneity of the dimer arrangement captured in different experimental contexts. Crucially, the local geometry around cleft “gate” residues central to our Site-2/2′ analysis remains well preserved (chain A: Cα deviations of 0.46–0.68 Å at His157/Tyr167/Thr263; local RMSD of 0.46–0.76 Å in residue-centered windows), validating the starting geometry for docking. Detailed superposition statistics (including the global RMSD, aligned Cα counts, per-protomer gate-residue deviations and local RMSDs) and the key-residue comparison table are provided in the Supplementary Materials (Table S5).

### 2.4. Global Stability of the ExcB–hSTING Corridor-Bound Complex over Three 100 ns Replicates

**Figure 2:**
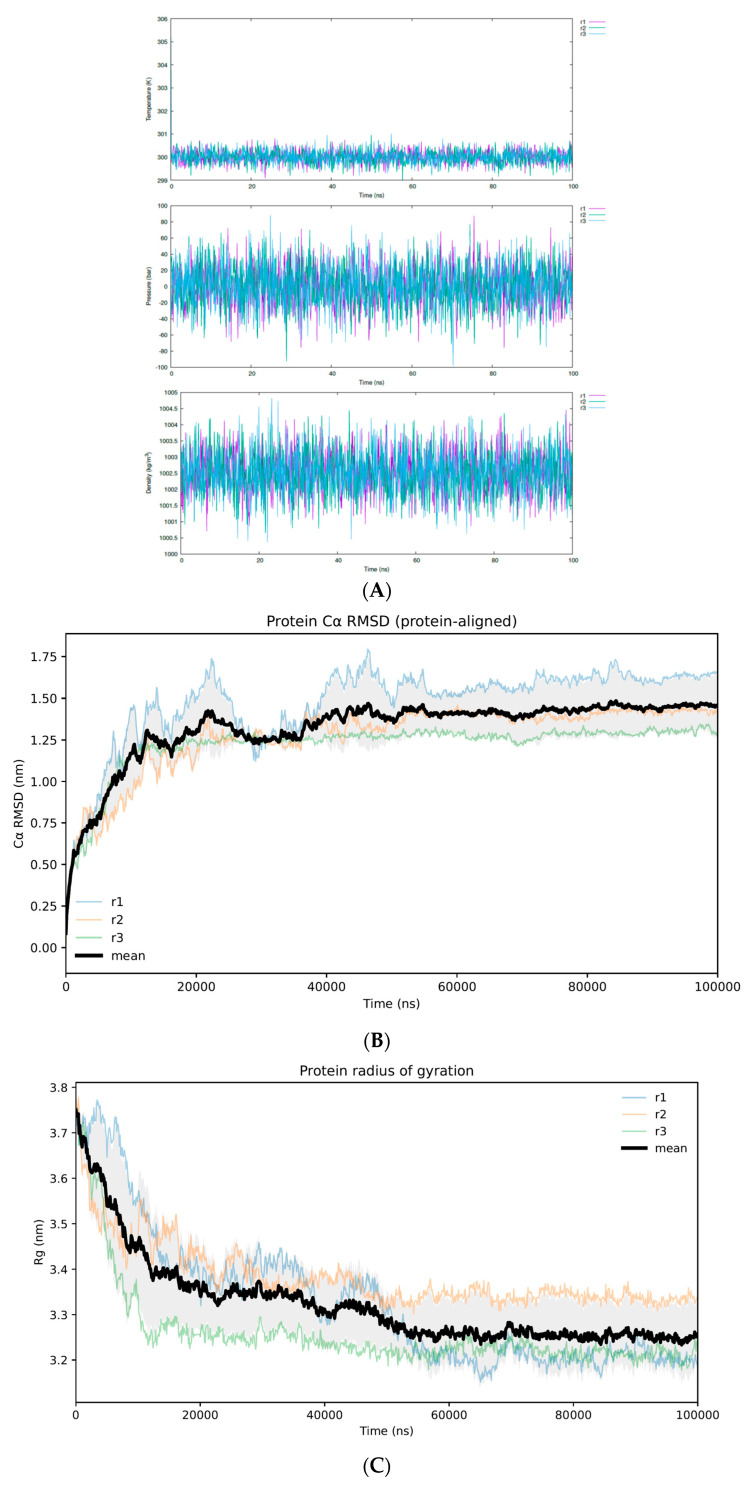

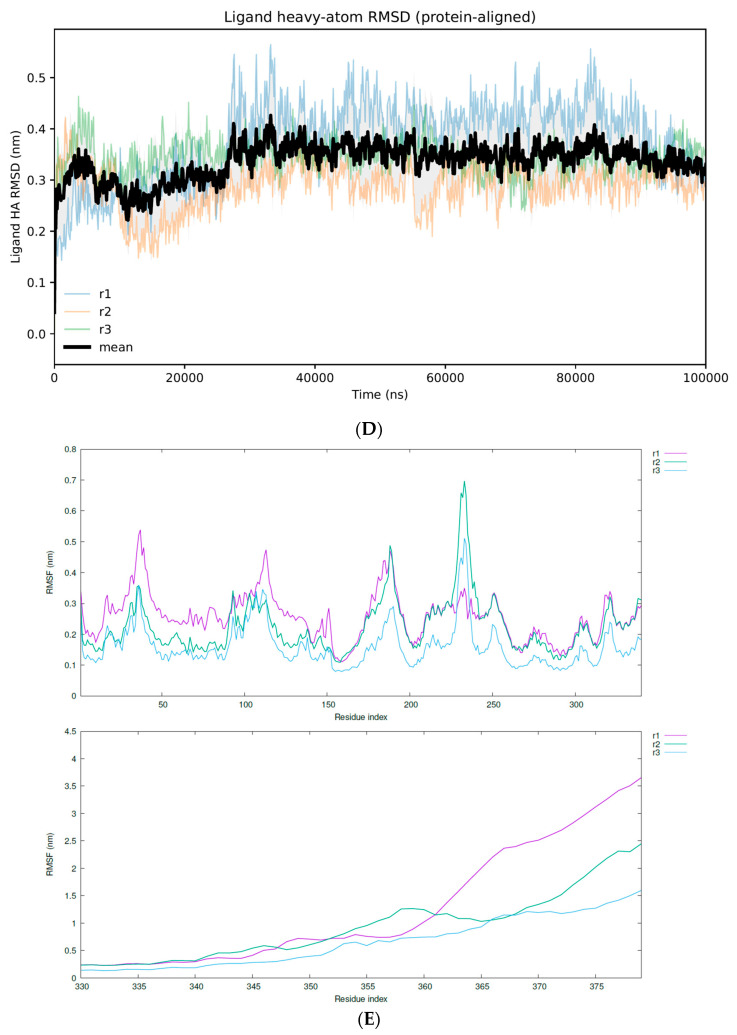
(**A**) System state points during production MD. Temperature, pressure, and solvent density are plotted as a function of simulation time for three independent 100 ns ExcB–hSTING trajectories (R1–R3). All replicas maintain stable NPT state points (T ≈ 300 K, P ≈ 1 bar, ρ ≈ 1000 kg·m^−3^), indicating robust thermodynamic control and no gross instabilities during the production runs. (**B**) Protein Cα RMSD (protein-aligned). Backbone Cα RMSD trajectories for the STING CTD dimer across the three independent replicas (R1–R3) after alignment to the protein, indicating overall fold stability over the simulation. (**C**) Protein radius of gyration (Rg). Time evolution of the protein radius of gyration for each replica, remaining essentially constant and suggesting the absence of global compaction/expansion events during the trajectories. (**D**) Ligand heavy-atom RMSD (protein-aligned). Protein-aligned ligand heavy-atom RMSD across replicas, reflecting the extent of ligand positional relaxation within the binding region while the receptor remains globally stable. (**E**) Residue-wise flexibility of the hSTING CTD dimer. Per-residue Cα RMSF (nm) computed from the three 100 ns replicas (R1–R3). RMSF is presented in a two-panel format to resolve the structured CTD core (upper panel) and a zoomed view of the highly mobile C-terminal tail region (residues ~330–375) (lower panel), highlighting localized flexibility without evidence of global destabilization.

To assess the stability of the Excavatolide B (ExcB)–hSTING complex in the Cys148-adjacent corridor, we conducted three independent 100 ns MD simulations under identical force-field settings but different initial velocity seeds (Replicas 1–3). System state points remained well maintained across all replicas, with temperature, pressure, and density fluctuating within expected ranges for a properly equilibrated NPT ensemble. This confirms stable thermodynamic control and the absence of gross instabilities during production trajectories (Figure 2A).

Backbone (Cα) RMSD profiles indicate that the hSTING CTD dimer remained conformationally stable throughout each 100 ns trajectory, without evidence of large-scale unfolding or progressive drift. In parallel, ligand heavy-atom RMSD trajectories stabilized at a plateau after an initial rise, reflecting the spatial progression from the entrance rim to the buried corridor. The plateaued RMSD profiles confirm the stable retention of ExcB within the corridor (Figure 2B–D). Consistent with this overall stability, the protein radius of gyration remained essentially constant over time, suggesting that global compaction/expansion events did not occur during the simulations (Figure 2C). Together, these global metrics indicate that the ExcB–hSTING complex samples a stable corridor-bound regime over the three replicas.

Residue-wise Cα RMSF further supported the stability of the structured CTD core while revealing localized flexibility in solvent-exposed regions. Across replicas, RMSF values remained low over most residues of the folded core, whereas increased mobility was primarily confined to loop regions and the C-terminal tail segment. To avoid compression of the core-scale fluctuations by the highly mobile tail, RMSF is shown in a two-panel format (Figure 2E). The tail exhibited substantially higher mobility and greater replica-to-replica variability, consistent with its nature as a flexible terminal segment outside the rigid CTD scaffold. This tail-localized flexibility does not confound the subsequent corridor-focused interaction analyses, which are driven by within-cavity contacts distinct from terminal dynamics.

### 2.5. Defining the Cys148–Adjacent Corridor (Site-2′) and Distinguishing It from Canonical Site-1 (Cys91)

To frame our analysis, we operationally define the Cys148–adjacent corridor (Site-2′) as a continuous, pocket-wall–guided pathway that originates at the CDN cleft rim (Site-2) and extends inward along the cleft wall. This geometry enables ExcB to adopt buried, within-cleft orientations while remaining non-covalently associated with the pocket. This definition serves as the geometric framework to classify within-cavity poses observed during MD and quantify subsequent residue-level contacts.

**Figure 3:**
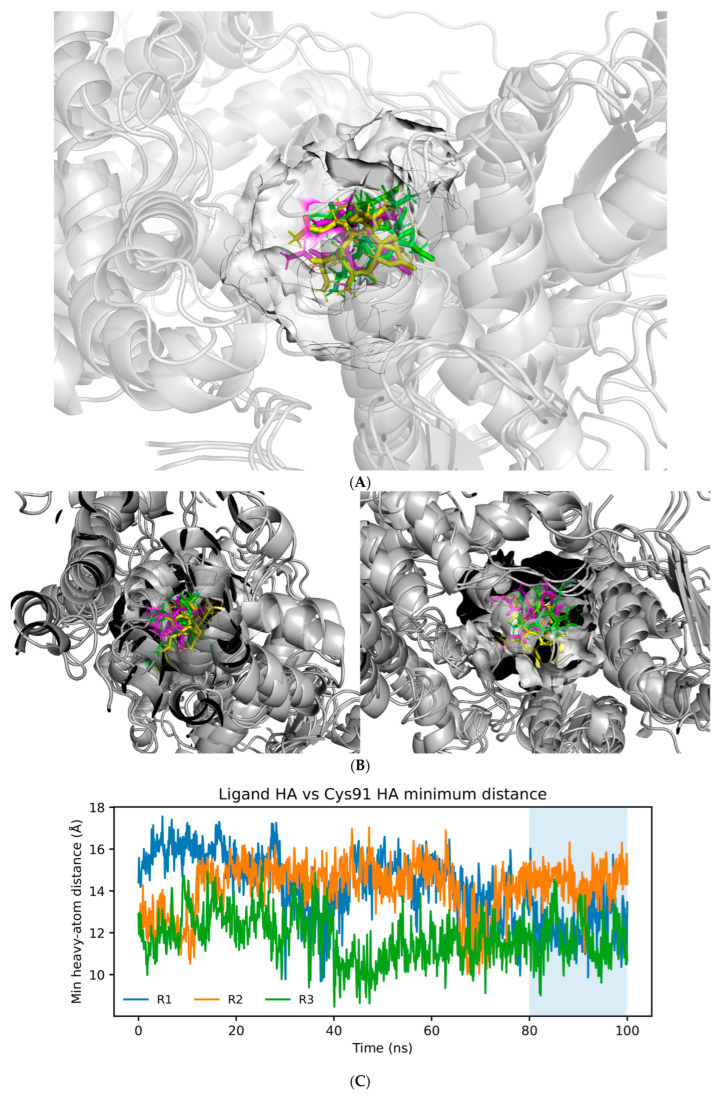

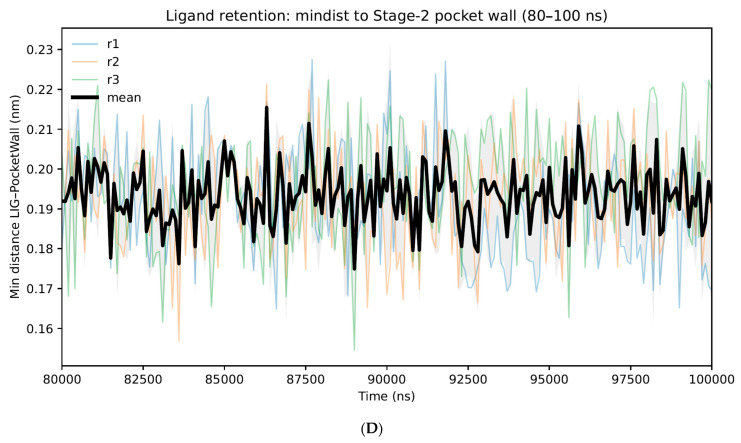
(**A**) Corridor confinement across replicas (surface view). Surface rendering of the hSTING CTD dimer highlighting the solvent-accessible CDN-binding cleft and the embedded corridor region. Representative ExcB conformations from three independent 100 ns replicas (snapshots near 90 ns) are overlaid as sticks, illustrating convergence into an overlapping corridor volume within the cleft. (**B**) Orthogonal views of the corridor-confined ensemble. Two rotated views (left and right) of the same snapshot overlay shown in (**A**), emphasizing that the three replica-derived ExcB conformations occupy a common corridor volume while differing subtly in local packing/orientation. (**C**) Cys91 exclusion quantified by ligand–Cys91 minimum distance. Time series of the minimum heavy-atom distance between ExcB and Cys91 across three replicas. The stabilized analysis window (80–100 ns) remains above the exclusion threshold (e.g., 3.5 Å), supporting a corridor interpretation distinct from the literature Site-1 (Cys91) regime. (**D**) Ligand retention quantified by minimum distance to the corridor pocket-wall set. Minimum distance between ExcB and the pocket-wall residue set that defines the corridor boundary, computed over 80–100 ns for each replica. Persistent short distances indicate continued corridor confinement during the stabilized window.

Two orthogonal analyses are consistent with this corridor model and its separation from the literature’s covalent Site-1 (Cys91) paradigm. First, pocket-centric visualization (surface rendering and pocket-wall residue mapping) shows ExcB sampling a confined, wall-guided path that progresses from the cleft mouth (Site-2) into a buried continuation consistent with the proposed corridor-like geometry (Figure 3A,B).

Second, distance-based analysis confirms that ExcB does not approach Cys91 within covalent-reactive range during stabilized trajectory segments. Consequently, Cys91 is excluded from the corridor residue set and from all “high-frequency contact” summaries reported below (Figure 3C). These results support a non-covalent, within-cleft corridor that is spatially and mechanistically distinct from the previously reported Cys91-adjacent Site-1 regime (Figure 3D).

### 2.6. High-Frequency Contact Residue Landscape Along the Cys148–Adjacent Corridor “Signature” Across Three 100 ns Replicas

To define the molecular boundary of ExcB occupancy, we next mapped the residue-level contact landscape within the Cys148-adjacent corridor. For each replicate, per-residue contact frequencies were computed using a uniform heavy-atom proximity criterion and aggregated over a stabilized sampling window (80–100 ns) to reduce sensitivity to early relaxation and transient entry sampling. Contacts were then compared across the three replicas to distinguish (i) consensus corridor residues that reproducibly define the corridor boundary from (ii) replicate-specific enrichments that reflect within-cavity microstate variability.

**Figure 4:**
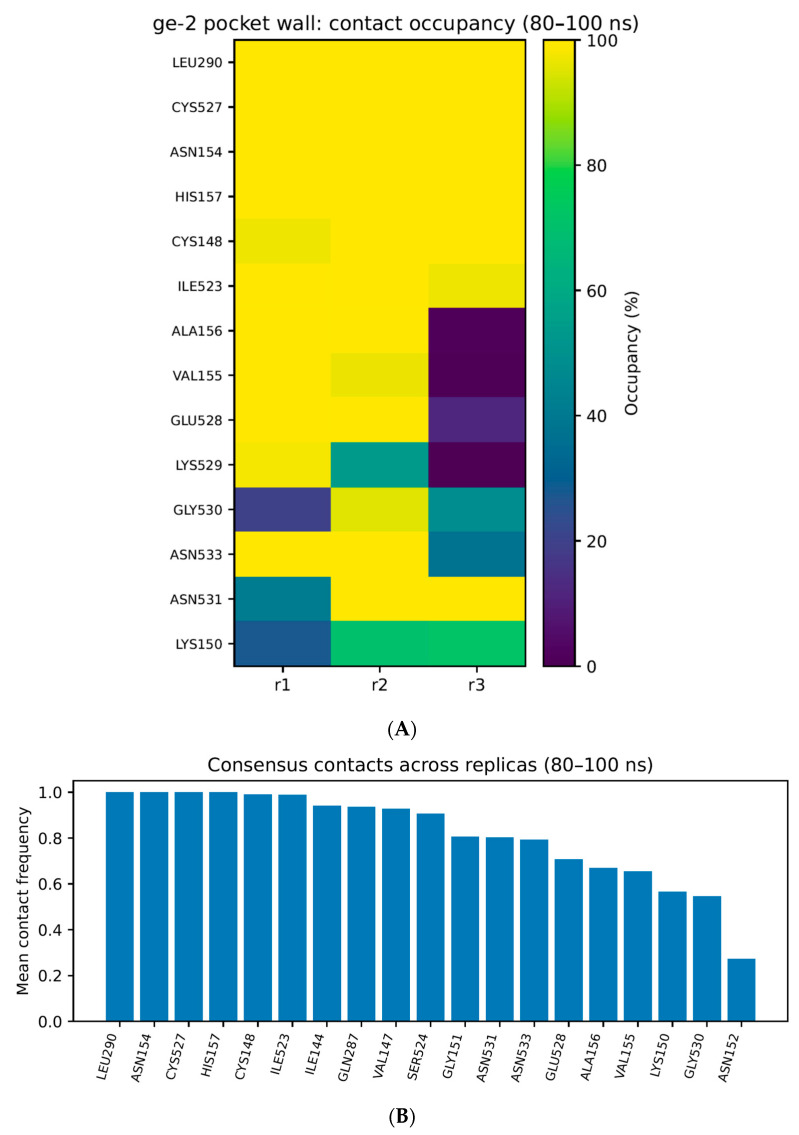
(**A**) Pocket-wall contact landscape defining the corridor geometry. Heatmap of per-residue contact frequency between ExcB and hSTING during the stabilized window (80–100 ns) across three independent replicas. A contact is counted when any ligand heavy atom approaches a residue heavy atom within 0.45 nm. High-frequency residues collectively delineate the corridor pocket-wall that confines ExcB. (**B**) Consensus high-frequency pocket-wall residues across replicas. Bar plot summarizing the consensus pocket-wall residues, defined as residues meeting the high-frequency criterion in ≥2 of 3 replicas, with bar heights representing contact frequencies in the stabilized window (80–100 ns). (A detailed residue list can be provided as Table S2 when available).

The resulting contact landscape identifies a defined set of high-frequency pocket-wall residues that collectively delineate the Site-2′ corridor geometry (Figure 4A).

To emphasize cross-replica reproducibility while avoiding over-interpretation of replica-specific fluctuations, we further report a consensus high-frequency set defined as residues meeting the frequency criterion in at least two of the three replicas (Figure 4B). This consensus set provides a conservative corridor “contact signature” that is robust to trajectory-to-trajectory microstate redistribution and is therefore the primary set emphasized in the main text. Notably, Cys91 is excluded from this high-frequency contact set, consistent with the distance-based exclusion established in Section 2.5 and further separating this mode from the covalent Site-1 mode.

For transparency, replica-specific contact-frequency bar plots are provided in the Supplementary Materials (Figure S3A–C) to document microstate-dependent enrichments without expanding the main-text figure set.

### 2.7. Within-Cavity Sub-Pose Diversity Within the Corridor: Stable vs. Transient Microstates Across Replicates

Although ExcB remains confined to the Cys148-adjacent corridor across three replicas of 100 ns, it does not maintain a single rigid pose. Instead, the trajectories indicate within-cavity sub-pose diversity. ExcB populates a limited number of corridor-restricted microstates that share the same macroscopic binding region but differ in orientation, burial depth, and pocket-wall contact fingerprints.

For practical interpretation, we classify these behaviors into two microstate classes:

(1) Stable microstates, which persist over extended trajectory segments and recur across replicates, exhibiting consistent corridor-wall contacts and sustained pocket confinement.
(2) Transient microstates, which appear as short-lived excursions within the corridor, redistributing local contacts without leaving the pocket-wall boundary.

Representative stabilized snapshots indicate that ExcB remains confined to an overlapping corridor volume across replicas. To quantify the spatial convergence of these microstates, we performed ligand-only GROMACS 2022.2 (Science for Life Laboratory, Stockholm, Sweden) clustering on the pooled 80–100 ns frames. As summarized in Table 1, the ensemble collapses into a single dominant cluster at a 0.15 nm cutoff (100%), while a stricter 0.10 nm cutoff resolves only a minor second cluster (2.0%). This confirms that the sub-pose diversity quantified by ligand-only clustering (Table 1) occurs within a spatially strictly confined volume. Thus, ExcB maintains a reproducible macroscopic binding mode while dynamically redistributing local interactions among corridor-wall residues.

### 2.8. Results—Gate Geometry and Cavity Descriptors (New/Replacement Subsection Text)

To quantify whether ExcB occupancy within the CDN-cleft region is accompanied by measurable changes in inter-protomer geometry, we tracked seven predefined A/B chain Cα–Cα distance pairs spanning the gate-adjacent region of the CTD dimer. These pairs were selected because (i) they lie on opposing protomers at the cleft rim and inner wall that define the effective “mouth” and internal corridor boundaries of the CDN-binding region, and (ii) they were consistently observable across all replicas with stable residue mapping, enabling direct first-vs-last window comparisons. For each replica, we computed mean distances over the full trajectory, the first 20 ns, and the last 20 ns, and then summarized the last-minus-first deltas to capture directional shifts in gate geometry while minimizing sensitivity to short-lived fluctuations (Supplementary Materials Table S7; see also the ranked gate-change statistics in Supplementary Materials Table S8).

Across replicas, several gate pairs exhibited directional changes consistent with a remodeled cleft geometry over time, while the magnitude of change varied between replicas. (Table S6) We interpret these metrics as observables of relative protomer geometry rather than as a single deterministic “cause” of ligand relocation; the results are therefore reported as replica distributions rather than over-interpreting any single trajectory. The replica-aggregated summary (mean ± SD across R1–R3) is provided to emphasize robustness of directionality where present and to transparently communicate variability where replicas diverge.

To characterize cavity dynamics in an analysis that does not presuppose a fixed pocket definition, we used mdpocket on the protein-only trajectories aligned on Cα atoms (output sampled every 2 ns). The resulting time series shows that cavity descriptors can diverge substantially across replicas (Figure S5), consistent with the known conformational plasticity of the STING CTD cleft. Rather than treating this as a contradiction, we treat replica spread as an expected feature of chaotic dynamics under identical thermodynamic and force-field settings. Accordingly, we report both the time-resolved trends (Figure S5) and the full per-snapshot descriptor table (Supplementary Materials Table S9) to enable transparent inspection and avoid over-claiming a single “true” volume trajectory. For a baseline comparison of these dynamic states against experimental structures, the calculated pocket volumes are summarized in Table 2. In the context of our mechanistic hypothesis, these results support a conservative statement: the CDN-cleft region samples multiple cavity states under MD, and ExcB can occupy a reproducible corridor-like microenvironment within this dynamic landscape, while the precise cavity volume trajectory is replica-dependent.

### 2.9. Competitive Docking of cGAMP on Native AF3 vs. ExcB-Conditioned MD Snapshots (~90 ns; 3 × 100 ns)

#### 2.9.1. Baseline Reference: cGAMP Docking to the Native AF3 Model

**Figure 5:**
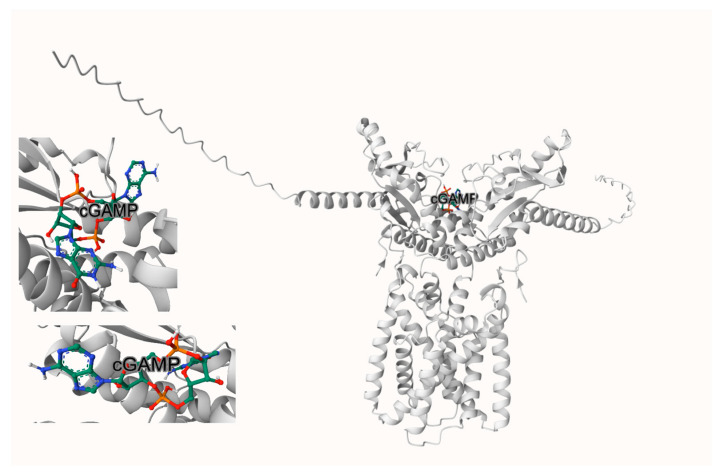
Native + cGAMP (entrance-open). Receptor shown in cartoon, cGAMP shown in sticks.

By docking cGAMP to the native (apo) hSTING, it yielded Vina scores of ~−10 to −11 kcal·mol^−1^ and adopted the canonical CDN-like pose in the cleft. This provides a baseline for productive accommodation and closure (Figure 5).

#### 2.9.2. Conditioned Receptor Compatibility: cGAMP Docking to Three ExcB Snapshots at ~90 ns

**Figure 6:**
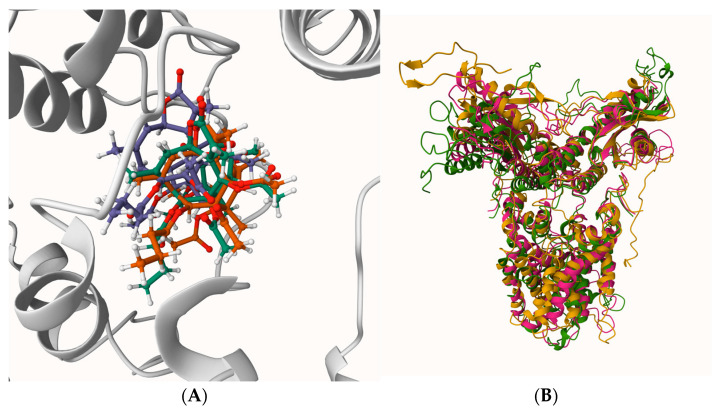
(**A**,**B**) Structural context of ExcB-conditioned STING snapshots (~90 ns). (**A**, **left**) Ligand-centered overlay of ExcB poses from three independent replicas (R1–R3) within the Site-2′ corridor after protein alignment, illustrating corridor-localized convergence of the ligand placement. (**B**, **right**) Global superposition of the corresponding receptor snapshots illustrating replica-to-replica conformational variability while retaining a broadly conserved central helical core.

To probe whether ExcB-conditioned STING conformations remain permissive to CDN engagement, we docked cGAMP to three receptor snapshots extracted at approximately 90 ns (one per independent MD replica). The structural context of these ExcB-conditioned snapshots involves corridor-localized ExcB poses and global replica-to-replica variability (Figure 6). Conditioned receptors were prepared by removing ExcB coordinates, and docking was performed using the same protocol and search definition as the native/open-state control (Section 4.4). For each conditioned receptor, we inspected the top five ranked poses for (i) occupancy of the canonical CDN-binding cleft and (ii) steric feasibility of the cleft-entering pose.

Docking to ExcB-conditioned receptor snapshots indicated a consistent qualitative trend toward reduced cGAMP cleft compatibility relative to the native/open reference under the same protocol. In replica R2, the top-ranked pose could be placed within the cleft without severe steric overlaps, yielding a favorable score of −7.5 kcal·mol^−1^. However, this value is notably weaker than the native/open-state docking score reported in Section 4.4 (range of −10 to −11 kcal·mol^−1^), suggesting a reduced docking favorability in the conditioned conformation even when in a sterically feasible pose.

In contrast, Replicas 1 and 3 (R1, R3) exhibited severe occlusion. The top-ranked solutions were predominantly displaced from the canonical cleft. Among the top five poses, only a single pose entered the cleft in each case, and these cleft-entering poses exhibited pronounced steric clashes (minimum heavy-atom separations of 1.86 Å for R1 and 0.87 Å for R3, with one to multiple close contacts under 2.0 Å). These metrics indicate that docking did not recover a physically plausible canonical pose within these conditioned clefts. (Table 3)

Taken together, these docking outcomes support a conformational conditioning mechanism, in which ExcB-conditioned STING conformations can become less accommodating to cGAMP in the canonical CDN-binding cleft. Because the binding penalty manifests even when cGAMP is docked without explicit co-occupancy by ExcB, the results are more consistent with receptor reshaping (an ExcB-induced structural deformation that reduces cleft permissiveness) rather than a purely steric, site-occupancy competition mechanism. While we avoid over-interpreting docking scores as absolute thermodynamic constants, the convergence of reduced affinity scores and prohibitive steric overlaps provides robust qualitative support for this non-competitive mechanism.

### 2.10. Docking-Workflow Benchmarking with Known Antagonists SN-011 and Astin C

To validate our computational pipeline prior to interpreting ExcB, we applied the identical receptor preparation, docking configuration, pose-selection criteria, and post-processing workflow to two reported human STING antagonists, SN-011 and Astin C [11,12]. Starting from the initial (0 ns) docking poses within the canonical CDN-binding cleft, we performed short explicit-solvent MD simulations (10 ns) to confirm that the docked complexes relax without ligand egress while retaining hallmark CDN-pocket interactions.

In both control trajectories, the ligands remained pocket-confined over the final 5 ns analysis window, as reflected by low within-window ligand RMSD, persistent ligand–protein proximity, and high atom-pair contact counts (Supplementary Table S4). Across the same window, SN-011 exhibited a slightly lower mean minimum ligand–protein distance and a higher mean contact count than Astin C, while Astin C remained comparably pocket-proximal. Representative top-ranked docking poses for the two controls are shown in Supplementary Materials Figure S2A,B.

### 2.11. In Silico ADMET and Developability

**Figure 7:**
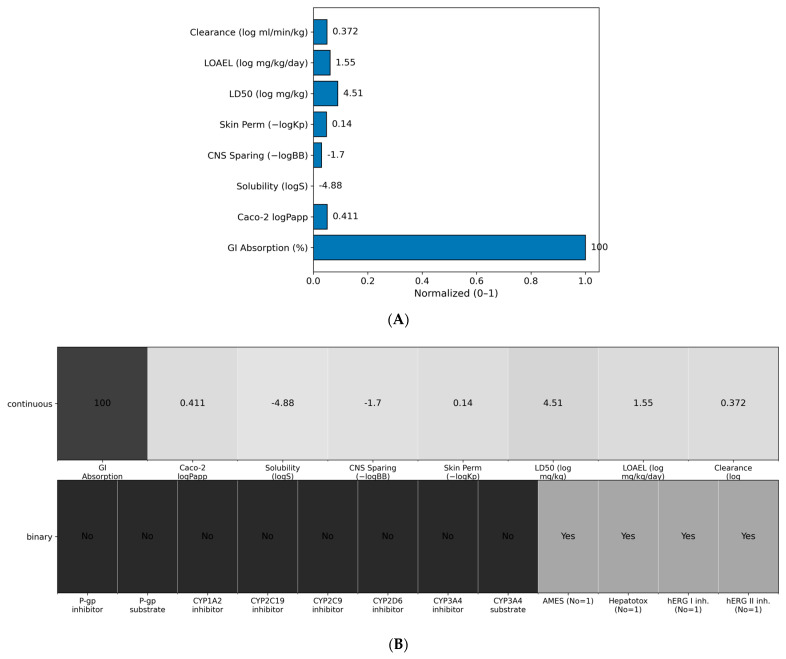
(**A**) Concise developability readouts for Excavatolide B. Continuous ADMET endpoints are shown as min–max–scaled bars (0–1) for visualization; numeric labels are the raw predictor outputs. Metrics (left → right): GI Absorption (%), Caco-2 logPapp, Solubility (logS), CNS sparing (–logBB), Skin permeability (–logKp), LD50 (log mg/kg), LOAEL (log mg/kg/day), and Clearance (log ml/min/kg). (**B**) Liability overview Top row repeats the continuous endpoints from (**A**) as a tile map; bottom row summarizes binary classifiers (P-gp inhibitor/substrate; CYP1A2/2C19/2C9/2D6/3A4 inhibitor/substrate; AMES, Hepatotoxicity, hERG I/II). Tiles aid readability only; interpretation should rely on the printed values/labels and the Supplementary Materials Table S5.

Aggregate in silico readouts for Excavatolide B (ExcB) indicate encouraging developability with tractable liabilities. pkCSM (University of Melbourne, Melbourne, Australia) predictions suggest high GI absorption (~100%), moderate permeability (Caco-2 logPapp ≈ 0.411), low aqueous solubility (logS ≈ −4.883), limited CNS penetration (−logBB ≈ 1.698), and low dermal permeability (−logKp ≈ 0.14). Predicted toxicity endpoints fall in moderate safety ranges (LD50 ≈ 10^4.51^ mg kg^−1^; LOAEL ≈ 10^1.546^ mg kg^−1^ day^−1^), with moderate clearance (log ml min^−1^ kg^−1^ ≈ 0.372). These continuous endpoints are summarized in Figure 7A and reported in Table S3.

Crucially, no binary liabilities were flagged by the models. Predictions were negative for P-gp inhibition/substrate status and for all CYP liabilities (CYP1A2/2C19/2C9/2D6/3A4 inhibitor and CYP3A4 substrate), suggesting minimal risk of transporter- or metabolism-mediated drug–drug interactions (DDI). Safety classifiers were also negative for genotoxicity (AMES), hepatotoxicity (pkCSM probability ≈ 0.12 for the “non-hepatotoxic” class), and cardiac risk (hERG I/II inhibition). These safety calls are shown in Figure 7B.

Overall, the in silico profile supports the feasibility of oral exposure, identifying aqueous solubility as the principal optimization lever while highlighting a clean toxicity and DDI safety profile.

## 3. Discussion

This study integrates AF3-guided receptor modeling, diffusion-based docking, and explicit-solvent molecular dynamics (MD) simulations to characterize the interaction between the briarane diterpenoid excavatolide B (ExcB) and the human STING cyclic dinucleotide (CDN)-binding cleft. Our results converge on a two-step inhibitory hypothesis: ExcB first samples a pose at the solvent-accessible rim of the cleft (Site-2), then relocates along a wall-guided pathway to populate a more embedded, non-canonical corridor within the cleft (Site-2′). This model is supported by convergence across three independent 100 ns MD replicas, which show stable protein fold metrics (RMSD, Rg) and ligand confinement to a reproducible corridor, distinct from the canonical covalent Site-1 (Cys91) binding mode reported in the literature.

The AF3-derived STING CTD dimer served as a starting receptor hypothesis and was benchmarked against representative experimental CTD structures (e.g., PDB: 4EF5, 6A05) using Cα-based comparisons. Because some experimental entries contain a single protomer in the asymmetric unit, we used the corresponding biological assembly when dimer-level geometry was required. We emphasize that these comparisons provide structural bounds for the starting fold and local gate-region geometry, rather than implying a uniquely correct dimer arrangement across experimental conditions. During simulations, ExcB displays a consistent spatial evolution from initial entrance sampling to stabilized corridor occupancy. In its entry mode (Site-2), ExcB partially encroaches on the canonical CDN-accessible volume, offering a plausible basis for direct competition under an open receptor geometry. However, the dominant behavior observed in the equilibrated phase of all replicas is occupancy of a buried corridor (Site-2′) on the Cys148-adjacent side of the cleft. Distance analyses confirm no persistent approach to Cys91, supporting a predominantly non-covalent, within-cleft mechanism.

A key mechanistic implication of the Site-2′ ensemble is the conformational conditioning of the cleft. Competitive docking of the native agonist cGAMP to receptor snapshots extracted from the late, ExcB-conditioned phase of the trajectories consistently yields less favorable scores compared to docking to the native baseline. Critically, the docking penalty persists even when ExcB coordinates are removed, indicating that the dominant penalty arises from receptor reshaping (“closed gate” conformation) rather than from persistent static steric clash with the ligand itself. This supports ExcB’s role as a conformational antagonist: while transient entrance sampling (Site-2) may disfavor cGAMP through local competition, corridor occupancy (Site-2′) biases the cleft toward a geometry that is intrinsically less accommodating to CDN recognition, often manifesting as prohibitive steric occlusion (as seen in R1 and R3) even in the absence of the modulator.

Residue-level analysis defines a reproducible corridor “contact signature” over the stabilized sampling window (80–100 ns). ExcB engages a compact set of high-frequency pocket-wall residues that delineate the corridor boundary. This landscape supports an ensemble of corridor-confined microstates rather than a single rigid pose; the ligand redistributes interaction preferences among neighboring wall residues, yielding sub-pose diversity while maintaining macroscopic confinement. This is mechanistically relevant, as allosteric antagonism can arise from an ensemble of related states that collectively bias the receptor away from an active conformation.

This two-step framework reconciles observations that might appear contradictory in isolation. Initial docking prioritizes the solvent-accessible cleft mouth (Site-2), whereas MD reveals the stabilization of a deeper, wall-guided corridor (Site-2′) that may not be the top-ranked cavity in static geometry-based analyses. Methodologically, we primarily report geometry-based contact frequencies to capture robust, replicate-consistent features, applying strict hydrogen-bond criteria only where explicitly stated to avoid over-interpreting transient polar interactions.

The proposed model is experimentally testable and offers clear design strategies. Mutagenesis of entrance-rim residues is predicted to modulate initial Site-2 sampling and competition, while perturbation of the high-frequency corridor-wall contact set should selectively affect buried occupancy and the degree of conformational conditioning. Biophysical probes, such as HDX-MS, NMR chemical-shift perturbation, or cavity volume measurements, should detect a less accessible or reshaped CDN cleft when the corridor ensemble is populated. Functionally, cellular assays can test whether ExcB induces a rightward shift in cGAMP dose–response curves, consistent with a conformational conditioning mechanism rather than acute, reversible competition alone.

Several limitations warrant consideration. Docking scores are not equivalent to binding free energies. MD sampling, while convergent across replicates, remains finite and is sensitive to force field parameters, protonation states, and initial conditions. The explicit modeling of water-mediated interactions and application of uniform hydrogen-bond criteria across trajectories would refine the residue-specific interaction picture. Nevertheless, the convergence of the corridor signature across independent replicates, coupled with the consistent weakening of cGAMP docking in ExcB-conditioned conformations, supports the proposed Site-2 → Site-2′ pathway as a coherent and testable structural hypothesis.

From a translational perspective, this mechanism outlines a non-covalent, cryptic-pocket strategy for STING antagonism. A corridor-confined, gate-closing mode offers a path to tunable antagonism that modulates CDN recognition through conformational bias, distinct from irreversible covalent inhibition. This provides a structure-based rationale for optimizing ExcB analogs, where chemical efforts can be directed toward enhancing either entrance sampling (for acute competition) or corridor stabilization (for sustained conformational conditioning), prioritizing the corridor-wall contact network for analog design. Physiologically, such a mechanism could allow graded suppression of pathological STING signaling, expanding the chemical space for safer, reversible modulators. The identified corridor contact signature and the conformational antagonism model thus nominate ExcB as a novel scaffold for probing within-cleft STING modulation and provide a defined set of residue-level hypotheses to guide subsequent medicinal chemistry and experimental validation.

## 4. Materials and Methods

### 4.1. AlphaFold3 Structural Modeling

The amino acid sequence of human Stimulator of Interferon Genes (hSTING) was obtained from the NCBI Protein Database (accession: NP_079510.1) in FASTA format. Structure prediction was performed using AF3, either through a local installation or a cloud-based environment (e.g., Google Colab) [13]. The resulting 3D structure was validated using predicted Local Distance Difference Test (pLDDT) scores to confirm model reliability.

To benchmark the AF3 hSTING CTD dimer against experimentally solved structures, we compared the relaxed AF3 model to representative human STING CTD crystal structures (PDB: 4EF5 and 6A05) by Cα-based structural superposition. The experimental structures were prepared by retaining the CTD dimer coordinates and removing non-protein components (ligands, waters, and crystallographic additives) where present. Superposition was performed in PyMOL 2.5 (Schrödinger, LLC, New York, NY, USA) (align/super; Cα atoms) using chain-consistent mapping (4EF5: A → A; 6A05: A → A and B → B). For each comparison we recorded (i) the global Cα RMSD and the number of common Cα atoms included in the fit, and (ii) local deviations at gate/pocket-defining residues central to the Site-2/Site-2′ analyses (His157, Tyr167, Thr263), quantified as per-residue Cα positional deviations and local-window Cα RMSDs (±5 residues around each site) for each protomer. The full superposition statistics and the key-residue comparison table were exported as a summary table and provided in the Supplementary Materials (Table S1).

### 4.2. Ligand Design and Optimization

The chemical structure of Excavatolide B (ExcB) was drawn using a chemical sketching tool (e.g., ChemDraw 25.5) and exported in SMILES format. RDKit was employed for 3D structure generation, followed by geometry optimization and energy minimization using Universal Force Field (UFF) or MMFF94 [14] to yield the lowest energy conformation suitable for docking.

### 4.3. Ligand Preparation and DiffDock Docking

The optimized hSTING and ExcB structures were processed using RDKit and Open Babel to convert ligand structures into 3D formats [15]. High-throughput docking was conducted using DiffDock, an AI-guided blind docking tool capable of rapid pose generation and scoring [16]. Output poses were evaluated based on spatial alignment and docking score, and top-ranked complexes were selected for further analysis.

### 4.4. GPU-Accelerated Docking Validation Using AutoDock Vina

Top docking poses from DiffDock were re-evaluated using AutoDock Vina (GPU-accelerated) for quantitative binding affinity scoring [17]. Ligands and hSTING were converted to the PDBQT format using AutoDockTools (ADT), with appropriate assignment of torsions, hydrogens, and atom types. Docking boxes were defined to encompass the predicted binding regions. GPU-accelerated docking simulations were executed to generate affinity values (kcal/mol) for each ligand-receptor pair. Post-docking visualization and qualitative interaction review were performed using PyMOL [18] and UCSF Chimera 1.19 [19].

### 4.5. Molecular Dynamics Simulations and Corridor/Contact-Frequency Analyses (3 × 100 ns)

#### 4.5.1. MD Engine and Force Field Setup

The hSTING CTD dimer was taken from the AF3 prediction and subjected to a brief relaxation (energy minimization followed by restrained equilibration) before production simulations [20]. ExcB was placed using the selected docking pose, and standard protonation/hydrogen placement was applied consistently across all systems. Protein parameters were assigned using an OPLS-AA family force field [21,22], while ExcB parameters were generated using LigParGen in an OPLS-compatible format [23]. The complex was solvated in explicit water [24] and neutralized with counter-ions. Additional NaCl was added where applicable to approximate physiological ionic strength.

#### 4.5.2. Energy Minimization and Equilibration Protocol

Each system underwent steepest-descent energy minimization to remove steric clashes, followed by restrained equilibration in two stages: (i) NVT equilibration to stabilize temperature and then (ii) NPT equilibration to stabilize pressure and density. During equilibration, positional restraints were applied to the protein heavy atoms to relax solvent/ions while preserving the initial binding geometry. Temperature control was maintained using a velocity-rescale thermostat [25] and pressure control using a Parrinello–Rahman barostat [26]. Short-range nonbonded interactions were calculated with a cutoff and Verlet neighbor lists [27], and long-range electrostatics were treated with PME [28]. Bond constraints were applied with LINCS [29], enabling a standard MD time step.

#### 4.5.3. Production MD Design: 3 Independent 100 ns Replicates

To assess robustness, three independent production trajectories (R1–R3) were performed for 100 ns each under identical settings, differing only in the initial velocity seed. Trajectories were saved at a uniform interval for subsequent analyses. Prior to analysis, trajectories were processed to remove periodic boundary artifacts and were aligned to the protein backbone to enable protein-referenced ligand metrics.

#### 4.5.4. Trajectory Processing and Global Stability Metrics

Protein backbone RMSD, residue-wise Cα RMSF, and radius of gyration (Rg) were computed to evaluate global stability of the CTD fold. Ligand heavy-atom RMSD was computed in the protein-aligned frame to quantify pocket retention while minimizing bias from global translation/rotation.

#### 4.5.5. Contact-Frequency Computation and High-Frequency Residue List Construction

Residue-level contact frequencies were computed using a heavy-atom proximity criterion: a protein residue was counted as contacting ExcB if any protein heavy atom lay within 0.45 nm (4.5 Å) of any ExcB heavy atom in a given frame. To reduce sensitivity to early relaxation, contact frequencies were summarized over a late, stabilized window (80–100 ns) for each replicate and then compared across replicas to identify consensus corridor residues versus replica-specific enrichments. The “corridor” (Site-2′) was operationally defined as the wall-guided within-cleft pathway contiguous with the cleft-rim entry (Site-2), as delineated by the reproducible high-frequency pocket-wall contact set. Distance-based readouts, including minimum distances to selected reference residues such as Cys91, were computed in parallel to separate corridor behavior from literature-reported Cys91-adjacent regimes. All contact-frequency source tables used for figure generation are summarized in Supplementary Materials Table S2. Distance- and hydrogen-bond diagnostics were computed in parallel as supportive readouts to distinguish corridor behavior from literature-reported Cys91-adjacent regimes (Supplementary Materials Figure S4).

### 4.6. Docking of cGAMP to ExcB-Conditioned STING Snapshots (~90 ns)

To assess cGAMP compatibility with ExcB-conditioned receptor conformations, three STING CTD dimer snapshots were extracted at ~90 ns from the three independent 100 ns MD replicas (R1–R3) of the ExcB–STING complex. For each snapshot, ExcB was removed and the receptor was prepared using the same settings as the native/open-state cGAMP docking control. cGAMP was docked using the identical protocol and search space as the native/open-state control used in Section 4.4. For each replica, the top five ranked poses were retained for analysis. Pose classification was based on (i) occupancy of the canonical CDN-binding cleft and (ii) steric feasibility, evaluated by minimum heavy-atom separation and the presence of severe close contacts (<2.0 Å) between cGAMP and receptor atoms.

### 4.7. Benchmark Controls with Known Antagonists (SN-011, Astin C): Docking + 10 ns MD

Docking setup. SN-011 and Astin C were prepared as 3D ligand structures and docked into the canonical CDN-binding cleft of hSTING using the same receptor preparation and docking configuration applied to ExcB. For each antagonist, the top-ranked pose consistent with canonical cleft occupancy was selected as the starting structure (“0 ns pose”) for short MD validation.

Short MD simulations. Each antagonist–hSTING complex was subjected to the same system-building procedure (solvation/ions/topology assembly) used for the ExcB simulations. Production MD was then run for 10 ns to verify pose stability and retention of canonical cleft contacts. The MD engine, force-field family, thermostat/barostat settings, electrostatics treatment (PME), constraints (LINCS), and trajectory processing steps were the same as described in Section 4.5, unless explicitly noted.

Readouts. Antagonist pocket retention was evaluated by protein-aligned ligand heavy-atom RMSD and by residue-level contact monitoring focused on the established CDN-pocket recognition network (e.g., Ser162/Tyr163/Tyr167/His232/Arg238). These controls were used strictly to benchmark the docking-to-short-MD workflow and to support the methodological credibility of the pipeline prior to application to ExcB.

### 4.8. ADMET Profiling

Complementing the mechanism, in silico ADMET (Absorption, Distribution, Metabolism, Excretion, Toxicity) was profiled to assess developability beyond target binding. Predictions were generated with ADMETlab 2.0 (Central South University, Changsha, China) [30], pkCSM [31], and ProTox-II [32] for oral absorption (e.g., Caco-2 permeability, P-glycoprotein interaction), systemic bioavailability, blood–brain barrier (BBB) penetration, plasma protein binding, CYP450 metabolism (CYP1A2, CYP2D6, CYP3A4), renal excretion, and toxicity endpoints (e.g., LD_50_, hERG inhibition, carcinogenicity).

### 4.9. Use of AI Tools

Use of AI Tools: A large language model (ChatGPT, model: 4.5-5.2, OpenAI, San Francisco, CA, USA) was used to assist with language polishing and minor structural editing of the manuscript. All scientific content, data analysis, and final wording were determined, checked, and approved by the authors.

## 5. Conclusions

This work supports a coherent, testable structural hypothesis for ExcB-mediated modulation of the hSTING CDN-binding cleft by integrating AF3-guided receptor modeling, docking, and explicit-solvent MD. Benchmarking of the AF3 hSTING CTD dimer against experimental structures (4EF5 and 6A05) supports the use of the AF3 model as a defensible starting point, particularly with respect to local gate/pocket geometry that frames the Site-2/Site-2′ analyses.

Across three independent 100 ns trajectories, ExcB consistently samples an entrance-proximal Site-2 at the cleft rim before relocating along a pocket-wall–guided path into a buried corridor (Site-2′) on the Cys148-adjacent side. Orthogonal distance/contact analyses do not support a persistent approach to the literature-reported covalent regime near Cys91, consistent with a predominantly non-covalent within-cleft mechanism.

In the stabilized sampling window, residue-level contact profiling defines a reproducible corridor “contact signature” while also revealing within-cavity sub-pose diversity, which is an ensemble of related microstates that remain corridor-confined yet redistribute interactions among neighboring pocket-wall residues.

Functionally, competitive docking controls indicate that ExcB-conditioned receptor conformations are less accommodating to cGAMP than the native/open receptor and that explicitly retaining ExcB does not further worsen cGAMP docking outcomes. This supports a conformational conditioning component rather than a purely steric blocking model.

Therefore, these findings outline a non-covalent, dynamics-sensitive (cryptic-pocket) strategy for STING antagonism and provide concrete experimental levers, including mutagenesis of entrance-rim and corridor-wall residues and biophysical/competition assays. These validate the proposed two-step Site-2 → Site-2′ pathway and prioritize ExcB analog optimization around the corridor contact network.

## Figures and Tables

**Table 1:** Ligand-only microstate clustering within the stabilized corridor window (80–100 ns, pooled).

Cutoff (nm)	Clusters	Dominant Cluster Occupancy	Minor Cluster Occupancy	Interpretation
0.15	1	603/603 (100%)	–	All frames fall into one cluster (highly consistent sub-pose).
0.10	2	591/603 (98.0%)	12/603 (2.0%)	Predominantly one cluster; rare geometric variant at stricter cutoff.

**Table 2:** Replica-aggregated gate-geometry shifts and mdpocket cavity descriptors for the STING CTD dimer trajectories (R1–R3). Caption (use in main text): Gate metrics report inter-protomer Cα–Cα distances (nm) for seven predefined pairs bordering the CDN-cleft region. Values are summarized as means over the first 20 ns and last 20 ns windows, with Δ defined as (last − first). mdpocket descriptors were computed from protein-only trajectories aligned on Cα atoms and sampled every 2 ns; the reported cavity descriptor(s) summarize the same first/last windows. Full per-replica time series and per-snapshot descriptors are provided in the Supplementary Materials (Figure S5; Tables S7–S9).

Replica	N_Snapshots	Pock_Volume_Mean	Pock_Volume_sd	Pock_Volume_Min	Pock_Volume_Max
R1	51	7851.031	1235.372	4693.11	9728.75
R2	51	4081.581	1005.29	2424.42	6394.17
R3	51	4653.014	594.1429	3561.54	6185.47

**Table 3:** cGAMP docking to ExcB-conditioned STING receptor snapshots (~90 ns). For each replica, the top five ranked poses were inspected and classified by whether they occupy the canonical CDN-binding cleft. Steric feasibility was assessed by the minimum heavy-atom distance between cGAMP and receptor atoms and by the number of cGAMP heavy atoms that exhibit a heavy-atom distance below 2.0 Å (clash count). Poses with severe overlaps were considered sterically infeasible. Docking scores are reported only as relative indicators under an identical protocol; our interpretation is based primarily on cleft occupancy and steric feasibility.

Replica (Snapshot ~90 ns)	R1	R2	R3
Best score (kcal·mol^−1^)	Unfavorable (positive)	Unfavorable (positive)	Unfavorable (positive)
poses in Top 5 located in canonical CDN cleft	1/5	1/5	1/5
Cleft-entering pose sterically feasible?	No	Yes	No
Min heavy-atom distance (Å)	1.86	2.97	0.87
Clash count (<2.0 Å) *	1	0	10
Qualitative outcome	Predominant off-cleft poses; the only cleft-entering pose shows steric overlap	Canonical cleft pose remains feasible with favorable score	Strong steric occlusion in the cleft; no valid pose found within the cleft

* Clash count: defined as the number of ligand heavy atoms with a distance below 2.0 Å to the receptor atoms.

## Data Availability

Details can be obtained by contact the authors directly.

## Supplementary Materials

The following Supplementary Materials can be downloaded at: https://www.mdpi.com/article/10.3390/ijms27052243/s1.

## Abbreviations

ADMET	Absorption, Distribution, Metabolism, Excretion, and Toxicity
AF3	AlphaFold3
BBB	Blood–brain barrier
CDN	Cyclic dinucleotide
cGAMP	2′,3′-cyclic GMP–AMP
cGAS	Cyclic GMP–AMP synthase
CNS	Central nervous system
CRC	Colorectal cancer (in context, colitis-associated colorectal cancer)
DDI	Drug–drug interaction
DiffDock	SE(3)-equivariant diffusion-based docking model
GI	Gastrointestinal
GROMACS	Groningen Machine for Chemical Simulations
HDX-MS	Hydrogen–deuterium exchange mass spectrometry
hERG	Human ether-à-go-go–related gene (KCNH2) potassium channel
hSTING	Human Stimulator of Interferon Genes
IFN-I	Type I interferon
IRF3	Interferon regulatory factor 3
LINCS	Linear Constraint Solver
LigParGen	Ligand Parameter Generator
LOAEL	Lowest observed adverse effect level
MD	Molecular dynamics
NF-κB	Nuclear factor kappa-light-chain-enhancer of activated B cells
NPT	Isothermal–isobaric ensemble (constant N, P, T)
NVT	Canonical ensemble (constant N, V, T)
OPLS-AA	Optimized Potentials for Liquid Simulations—All-Atom
PDB	Protein Data Bank
PDBQT	PDB format with partial charges and AutoDock atom types
PME	Particle-mesh Ewald
ProTox-II	In silico toxicity prediction web server
PyMOL	The PyMOL Molecular Graphics System
R_g	Radius of gyration
RMSD	Root-mean-square deviation
RMSF	Root-mean-square fluctuation
STING	Stimulator of Interferon Genes
TBK1	TANK-binding kinase 1
TME	Tumor microenvironment
UCSF Chimera	Molecular visualization system
Vina	AutoDock Vina docking engine
v-rescale	Velocity-rescale thermostat
