# Supplementary material for: Computational Analysis of Excavatolide B–Human STING Interactions Implicates a Cys148–Adjacent Corridor with Within-Cavity Sub-Pose Diversity
Source: Int J Mol Sci. 2026 Feb 27;27(5):2243. doi: 10.3390/ijms27052243 (PMC12984402; doi:10.3390/ijms27052243)

Supplementary Information (SI)

Computational analysis of Excavatolide B–human STING interactions implicates a Cys148–adjacent corridor with within-cavity sub-pose diversity

---

S-AF3. Receptor model confidence (AlphaFold3)

- Run mode: Template-free (no template block in JSON; no template library).
- Confidence metrics: Model-wide pLDDT ≈ 77; chain-wise ≈ 77/77 (A/B).
- Gate residues: His157 ≈ 90, Tyr167 ≈ 89, Thr263 ≈ 88 (per-atom pLDDT from mmCIF B-factor).
- Interface: ipTM = 0.79, pTM = 0.80; inter-protomer PAE mean ≈ 13.6 Å (median ≈ 9.7 Å; P10--P90 ≈ 4.4--31.1 Å).
- Relaxation: Short minimization + restrained equilibration; gate/pocket geometry unchanged.

Full panels are now provided as Figure S1A (PAE map) and Figure S1B (per-residue pLDDT) to allow localized confidence inspection at the gate/pocket.

S-AF3-VAL. AF3 benchmarking against experimental STING CTD structures (Table S1 + Data S1).

The relaxed AF3 hSTING CTD dimer model was benchmarked against representative experimental CTD structures (PDB: 4EF5, 6A05) using Cα-based structural superposition with chain-consistent mapping. The full exported alignment summary (global Cα RMSD, number of common Cα atoms, and local deviations at gate residues His157, Tyr167, and Thr263) is provided as **Table S1**.

S-DOCK-CTRL. Benchmark docking controls (SN-011 and Astin C).

To contextualize the ExcB docking/MD analyses and provide positive controls for the docking-to-MD workflow under the same modeling conditions, we docked two reported STING antagonists (SN-011 and Astin C) into the same relaxed AF3 STING CTD dimer model. Receptor preparation, grid definition centered on the CDN mouth/rim region, and docking workflow were kept consistent with those used for

the main ExcB and cGAMP docking analyses. The top-ranked poses localize to the solvent-accessible rim/entrance of the CDN-binding cleft, providing a qualitative sanity check that the adopted docking setup can recover entrance-proximal binding modes for known antagonists. These controls are included for spatial reference and standardized visualization; they are not used to infer relative potency or absolute binding free energies. Representative top-ranked poses for SN-011 and Astin C are shown in **Figure S2A,B**.

To complement the docking snapshots, short-timescale MD stability diagnostics were computed for both control systems. A summary of the final 5 ns window, including ligand RMSD, ligand–protein minimum distance, and atom-pair contact counts, is provided in **Supplementary Table S4**. Together with the representative docking poses, these controls support that the adopted docking-to-MD workflow yields pocket-confined and stable antagonist poses under the same modeling conditions.

S-CONTACT. Corridor contact-frequency source tables (80–100 ns; r1–r3) (Figure S3A-C, Table S2).

To identify residues that reproducibly contact ExcB within the corridor during the converged simulation segment, we computed per-residue contact frequencies for the 80–100 ns window independently for each 100 ns replicate (r1–r3). Per-replicate contact-frequency distributions are shown in **Figure S3A–C**. A consensus residue table is provided as **Table S2**, reporting per-replicate frequencies and replicate counts (≥2/3 replicas) with a functional annotation of each residue's positional role (rim/wall/gate).

S-DIST-HB. Distance- and hydrogen-bond diagnostics (r1–r3). (Figure S4).

Distance- and hydrogen-bond-based diagnostics were computed as orthogonal readouts to support interpretation of corridor confinement across replicas. Specifically, we report (i) the minimum heavy-atom distance between ExcB and His157 (panel A), (ii) center-of-mass (COM) distances between ExcB heavy atoms and the gate residues His157, Tyr167, and Thr263 (panels B-D), and (iii) the protein-ligand hydrogen-bond counts over time (panel E). These traces are intended as supportive diagnostics for pocket confinement and gate engagement; they are not used as binding free-energy surrogates.

S-ADMET. Cross-tool ADMET table and notes.

We provide full, tool-native ADMET outputs (SwissADME, ADMETlab3; pkCSM when applicable). Rows and columns are left unaltered; no cross-tool averaging or voting is performed.

Files: ADMET_consensus.csv (concise endpoints), ADMET_merged_wide.csv (wide merge of all columns).

Reported endpoints: MW, TPSA, Consensus LogP, ESOL LogS/class; GI absorption (SwissADME), HIA probability (ADMETlab3), BBB probability; P-gp inhibitor/substrate; CYP1A2/2C19/2C9/2D6/3A4 inhibition/substrate; hERG I/II; AMES; DILI; clearance; bioavailability metrics (SwissADME score; ADMETlab f20/f30).

Interpretation caveats: Endpoint definitions, training data, thresholds, and units can differ. Hence, apparent disagreements are expected and should not be force-resolved computationally.

Minimal wet-lab verification plan:

- Permeability (Caco-2 or PAMPA).
- Metabolic stability and CYP inhibition (HLM/CYP panel).
- Cardiotoxicity risk (hERG surrogate).
- Genotoxicity/hepatotoxicity screens (Ames/DILI surrogates).

Supplementary Table S1. AF3-to-crystal STING CTD benchmarking summary (Cα superposition metrics and gate-residue deviations).

comparison	chain_map	global_RMSD_A	n_common_CA	af3_chain	d157_A	d167_A	d263_A	localRMSD_157win_A	localRMSD_167win_A	localRMSD_263win_A
AF3_vs_4EF5	A->A	1.032	177	A	0.643	0.447	0.151	2.299	0.368	0.257
AF3_vs_6A05	A->A; B->B	5.305	364	A	0.459	0.681	0.615	0.76	0.703	0.459
AF3_vs_6A05	A->A; B->B	5.305	364	B	1.329	2.865	3.256	1.47	3.226	4.225

Supplementary Table S2. Consensus corridor-contact residues for ExcB across three independent 100 ns replicas (contact frequencies over the 80–100 ns window).

Residue	resid	resname	replicate_count	r1	r2	r3	mean	sd	Role
LEU290	290	LEU	3	100	100	100	100	0	wall
CYS527	527	CYS	3	100	100	100	100	0	wall
ASN154	154	ASN	3	100	100	100	100	0	wall
HIS157	157	HIS	3	100	100	100	100	0	wall
CYS148	148	CYS	3	97.0149	100	100	99.00497	1.723448	wall
ILE523	523	ILE	3	100	99.5025	97.0149	98.83913	1.599296	wall
ASN531	531	ASN	3	40.796	100	100	80.26533	34.18145	rim/variable
ASN533	533	ASN	3	100	100	37.8109	79.2703	35.90489	rim/variable
GLU528	528	GLU	3	99.5025	100	12.4378	70.64677	50.41106	rim/variable
LYS150	150	LYS	3	27.8607	70.1493	71.6418	56.5506	24.85739	rim/variable
GLY530	530	GLY	3	19.9005	95.0249	48.7562	54.56053	37.89705	rim/variable
ALA156	156	ALA	2	99.5025	100	1.4925	66.99833	56.73026	rim/variable
VAL155	155	VAL	2	99.5025	96.5174	0.4975	65.5058	56.31862	rim/variable
LYS529	529	LYS	2	98.01	53.7313	0	50.58043	49.08091	rim/variable

Supplementary Table S3. ADMET

Category	Endpoint	Unit	SwissADME	ADMETlab 3.0
Physicochemical	Molecular formula		C30H42O12	
Physicochemical	Molecular weight	g/mol	594.65	594.27
Physicochemical	Topological polar surface area (TPSA)	Å²	164.26	164.26
Physicochemical	H-bond acceptors (HBA)	count	12	12
Physicochemical	H-bond donors (HBD)	count	1	1
Physicochemical	Rotatable bonds	count	10	10
Physicochemical	Fraction Csp3		0.77	0.767
Physicochemical	logP (octanol/water)		2.5	1.985
Physicochemical	logD (octanol/water)			2.231
Physicochemical	logS (aqueous solubility)		-4.23	-3.761
Physicochemical	pKa (acidic)			6.647
Physicochemical	pKa (basic)			3.848
Physicochemical	Melting point	degree C		189.149
Physicochemical	Boiling point	degree C		301.648
Physicochemical	Bioavailability score		0.17	
Physicochemical	Synthetic accessibility		7.69	6

Solubility (SwissADME models)	ESOL solubility	mg/mL	0.035	
Solubility (SwissADME models)	Ali solubility	mg/mL	0.003	
Solubility (SwissADME models)	Silicos-IT solubility	mg/mL	0.263	
Solubility (SwissADME models)	Ali logS		-5.25	
Solubility (SwissADME models)	Silicos-IT logSw		-3.35	
Absorption	Caco-2 permeability	log unit		-5.053
Absorption	MDCK permeability	log unit		-4.763
Absorption	PAMPA (Peff)	logPeff		0.965
Absorption	Skin permeation (Kp)	log10(cm/s)	-8.39	
Distribution	Plasma protein binding (PPB)	%		56.923
Distribution	Fraction unbound (Fu)	%		36.558
Distribution	Volume of distribution (VDss)	log10(L/kg)		-0.555
Distribution	BBB+ probability	%		0.004
Excretion	Plasma clearance (CLplasma)	mL/min/kg		5.863
Excretion	Half-life (T1/2)	h		1.063

Supplementary Table S4. Final 5 ns MD stability summary for SN-011 and Astin C control systems.

Values summarize the final 5 ns analysis window (501 frames) of the control trajectories, including within-window ligand RMSD, minimum ligand–protein distance, and atom-pair contact counts (<0.45 nm). These metrics are used to assess pocket confinement and relative proximity of the two control antagonists.

SYS	metric	N	mean	sd	min	max
AstinC	rmsd_prot_win	501	0.259871	0.017304	0.216718	0.310279
AstinC	rmsd_lig_win	501	0.335081	0.0889	0.152527	0.480283
AstinC	rg_prot_win	501	3.158738	0.008513	3.13472	3.18406
AstinC	mindist_LIG_PROT_win	501	0.211348	0.010979	0.163725	0.235037
SN-011	rmsd_prot_win	501	0.248505	0.016006	0.211402	0.289965
SN-011	rmsd_lig_win	501	0.310722	0.024852	0.230657	0.386832
SN-011	rg_prot_win	501	3.198656	0.009008	3.17072	3.22338
SN-011	mindist_LIG_PROT_win	501	0.200741	0.014942	0.160327	0.238036

AstinC	ncont_atomPairs_lt0.45nm	501	690.725	47.519	565	850
SN-011	ncont_atomPairs_lt0.45nm	501	747.078	36.174	630	866

Supplementary Table S5. Gate-pair distances in an experimental hSTING CTD structure (PDB: 6A05) for the seven selected inter-chain residue pairs.

To provide an experimental structural bound for the "gate-region" geometry used in our MD-based analyses, we extracted the inter-chain distances for the seven gate pairs (A154–B154, A154–B161, A157–B157, A287–B161, A287–B287, A290–B161, A290–B290) from the experimentally determined hSTING CTD structure (PDB: 6A05). These pairs were chosen because they span the CDN-cleft rim-to-wall "mouth/gate" region adjacent to the Cys148-side corridor and directly report relative A/B chain positioning around the entrance/corridor boundary. Values are reported in nm and used as a conservative reference for the starting geometry and for interpreting the magnitude of MD-observed gate changes (Table S7) without over-claiming a static "corridor" in any single PDB snapshot.

pair	value_nm
6A05_A154_B154	0.741
6A05_A154_B161	1.021
6A05_A157_B157	0.961
6A05_A287_B161	1.178
6A05_A287_B287	1.762
6A05_A290_B161	1.568
6A05_A290_B290	1.872

Supplementary Table S6. Ranked gate-associated residues by aggregate gate-change magnitude across replicas (derived from Table S7).

We aggregated the absolute gate-change magnitudes (|Δ|, in Å) across all seven gate pairs and all replicas (Table S7) and mapped each pairwise change to its two constituent residues (e.g., A290–B290 contributes to A290 and B290). For each residue, we report the number of contributing pairs (n_pairs), the summed absolute change across those pairs (sum_abs_delta_A), and the mean absolute change per pair (mean_abs_delta_A). This ranking provides a practical prioritization of corridor-

/gate-proximal residues for hypothesis-driven experimental perturbation (e.g., mutagenesis), while remaining conservative: the ranking reflects geometric sensitivity around the cleft mouth/corridor boundary, not a claim of direct biochemical causality.

residue_id	n_pairs	sum_abs_delta_A	mean_abs_delta_A
A290	6	126.242	21.04
A287	6	120.905	20.151
B161	9	96.117	10.68
B290	3	94.139	31.38
B287	3	90.146	30.049
A154	6	58.629	9.771
A157	3	54.484	18.161
B157	3	54.484	18.161
B154	3	25.374	8.458

Supplementary Table S7. Gate-pair distance statistics over early vs late trajectory windows across three independent 100 ns replicas.

For each replica (R1–R3) and each of the seven gate pairs, we report mean distances over the full trajectory (mean_all_nm), the first 20 ns window (mean_first20ns_nm), and the last 20 ns window (mean_last20ns_nm), together with the difference between late and early windows (Δ = last20 − first20) in nm and in Å (delta_Ang). These metrics quantify how the A/B chain relationship around the CDN cleft mouth/gate evolves during MD. Across replicas, the direction and magnitude of changes can differ (reflecting the stochastic sensitivity of chaotic dynamics under different initial velocities), so we report per-replica values explicitly and avoid over-interpreting any single trajectory.

replica	pair	mean_all_nm	mean_first20ns_nm	mean_last20ns_nm	delta_last_minus_first_nm	delta_Ang
R1	A154-B154	10.53589	10.84647	9.152851	-1.69362	-16.936
R1	A154-B161	9.826528	9.005473	7.516065	-1.48941	-14.894
R1	A157-B157	11.06292	11.97463	11.17912	-0.79551	-7.955
R1	A287-B161	9.858117	8.985179	7.668642	-1.31654	-13.165
R1	A287-B287	11.25392	10.72301	11.84575	1.122746	11.227

R1	A290-B161	9.840208	8.977786	7.587473	-1.39031	-13.903
R1	A290-B290	11.02078	11.81313	9.770443	-2.04269	-20.427
R2	A154-B154	11.00744	10.76098	10.06774	-0.69324	-6.932
R2	A154-B161	11.32637	9.429095	10.62893	1.19983	11.998
R2	A157-B157	9.61842	11.84768	7.777269	-4.07041	-40.704
R2	A287-B161	11.36744	9.494562	10.57186	1.077299	10.773
R2	A287-B287	10.25913	12.17759	6.447498	-5.73009	-57.301
R2	A290-B161	11.34369	9.452871	10.60893	1.156059	11.561
R2	A290-B290	11.42587	13.1319	10.46277	-2.66913	-26.691
R3	A154-B154	10.68268	10.95037	11.10094	0.150567	1.506
R3	A154-B161	10.40671	11.04492	10.40866	-0.63625	-6.363
R3	A157-B157	10.89549	11.57383	10.99137	-0.58246	-5.825
R3	A287-B161	10.42975	11.03715	10.35507	-0.68208	-6.821
R3	A287-B287	11.38984	13.90071	11.73894	-2.16177	-21.618
R3	A290-B161	10.41072	11.034	10.3701	-0.66389	-6.639
R3	A290-B290	9.782967	13.30265	8.600527	-4.70212	-47.021

Supplementary Table S8. mdpocket pocket-volume summary statistics across replicas (2 ns sampling; protein-fit trajectories).

Pocket volumes were computed using mdpocket on protein-only, Cα-fitted trajectories sampled every 2 ns (51 snapshots over 0–100 ns). For each replica we report the number of snapshots analyzed and the mean/min/max of the mdpocket-reported pocket volume (Å^3). These values summarize the replica-to-replica spread and provide quantitative context for the time-resolved pocket-volume trace shown in Figure S5. Importantly, this analysis reports an “accessible pocket volume” defined by mdpocket’s geometric detection on the fitted protein ensemble; it is a structural descriptor rather than a binding free-energy estimate.

replica	n_snapshots	pock_volume_mean	pock_volume_min	pock_volume_max
R1	51	7851.031	4693.11	9728.75
R2	51	4081.581	2424.42	6394.17
R3	51	4653.014	3561.54	6185.47

Supplementary Table S9. Full mdpocket descriptor table for all snapshots across R1–R3 (2 ns sampling).

This table contains the complete mdpocket descriptor output for each analyzed snapshot (1–51 per replica), including pocket volume (pock_volume), solvent-accessible surface area (pock_asa), polar/apolar ASA partitions, convex hull volume, and residue-type counts contributing to the detected pocket. Rows are concatenated across replicas (R1–R3) to enable downstream plotting and summary statistics (e.g., Table S8 and Figure S5). The descriptor definitions follow mdpocket/fpocket conventions; no rescaling or post hoc normalization was applied beyond concatenation and consistent snapshot indexing.

Figure S1. AlphaFold3 Predicted Alignment Error (PAE) heatmap and per-atom pLDDT distribution for the human STING dimer.

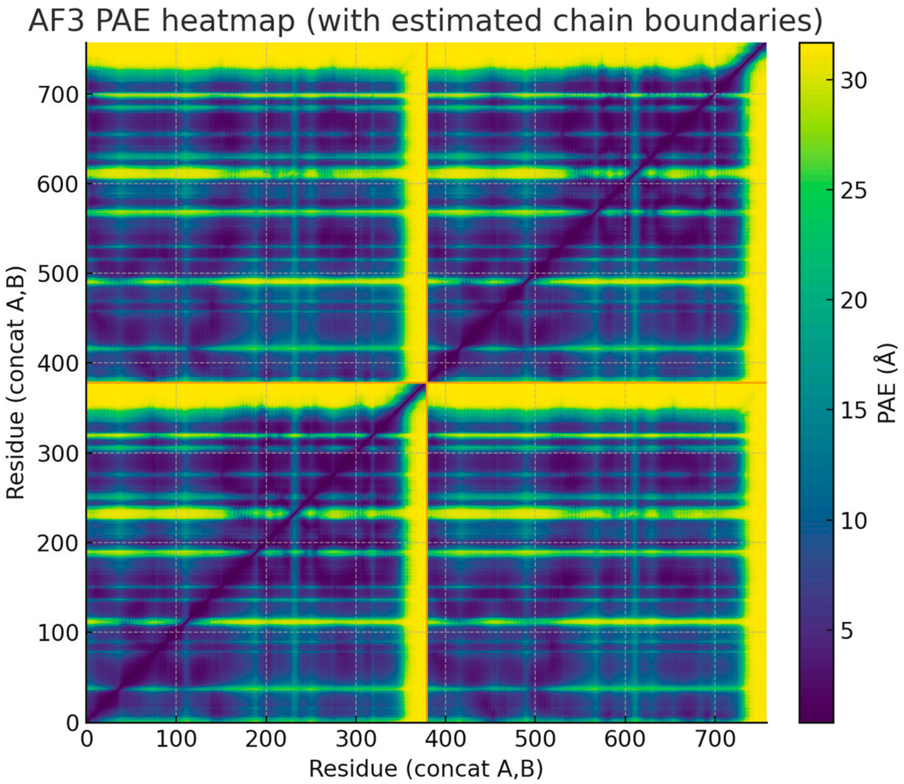

Figure S1A.

PAE values (Å) are shown for the AF3 dimer model as a residue–residue matrix after concatenating chain A (0–N) and chain B (N–2N). Lower values (purple) indicate higher confidence in relative placement; yellow indicates high uncertainty. Dashed lines mark the estimated chain boundaries and the ligand-proximal "mouth" region. This panel supports the localized confidence around the CDN rim used for docking and MD initialization.

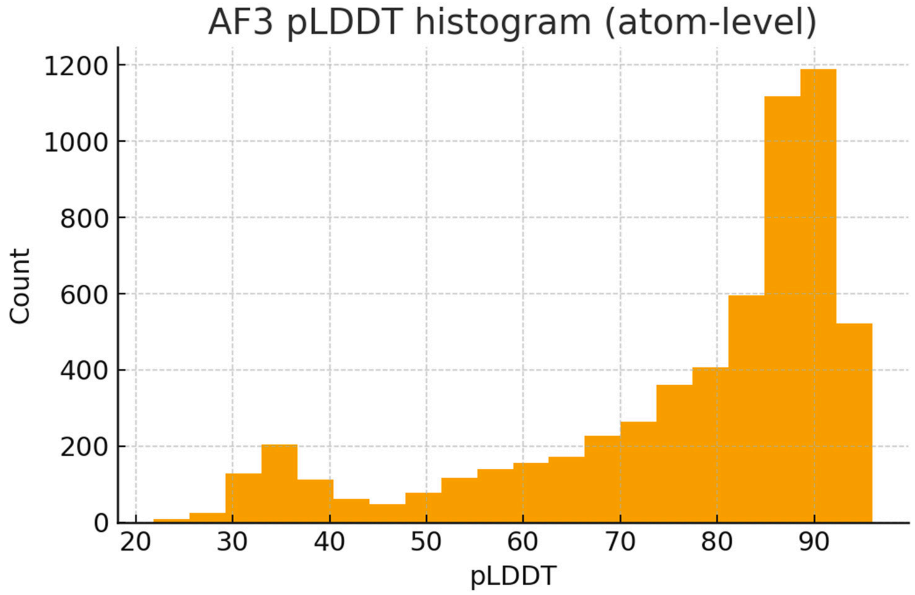

Figure S1B. Histogram of per-atom pLDDT scores for the AF3 dimer model. Scores >90 denote very high local confidence, 70–90 high, 50–70 low, and <50 very low. The majority of pocket and rim atoms fall in the high-confidence regime, consistent with using this model for docking and short MD.

Figure S2. Benchmark control: SN-011 and Astin C docking into the AF3 STING dimer (top-ranked Vina-GPU poses near the CDN mouth).

Docking-only controls are shown; poses are top-ranked under the same scoring function and search space used in the main docking analyses.

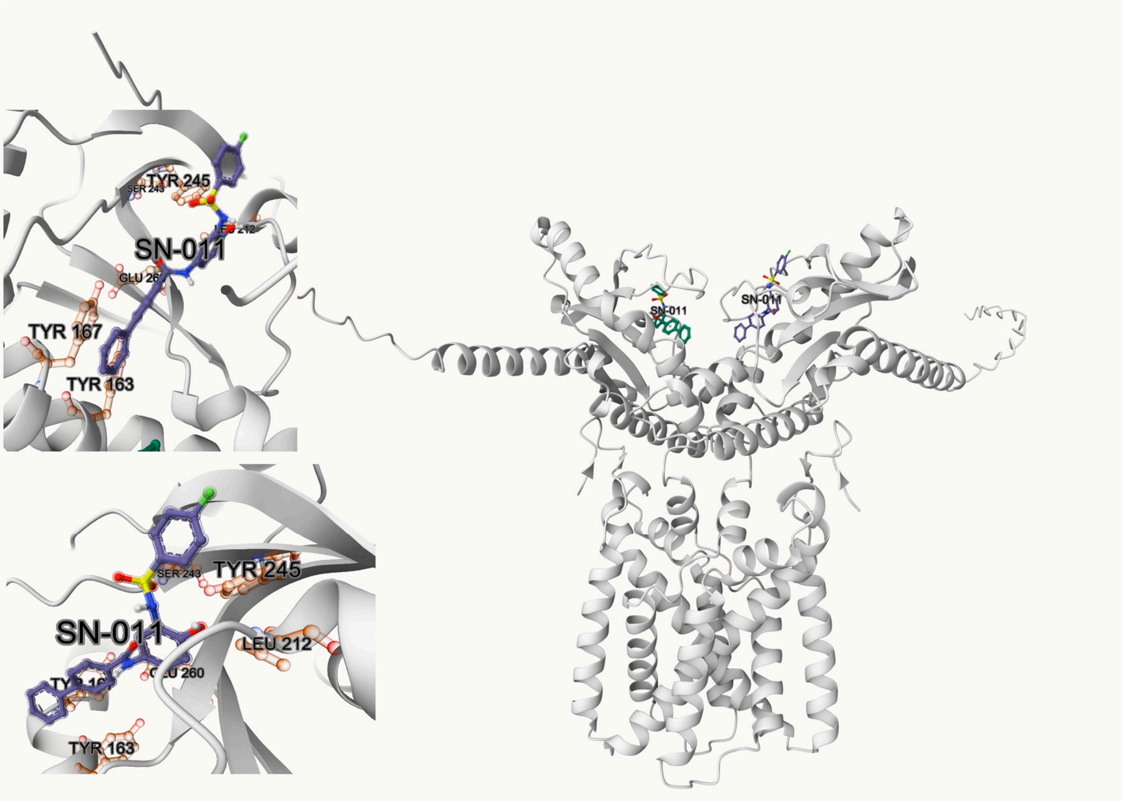

・Figure S2A. Two top-ranked poses of SN-011 are shown near the CDN entrance (sticks; protein in gray cartoon; key pocket residues as sticks with semi-transparent surfaces). The panel demonstrates canonical Site-2 engagement consistent with literature-reported antagonist contacts.

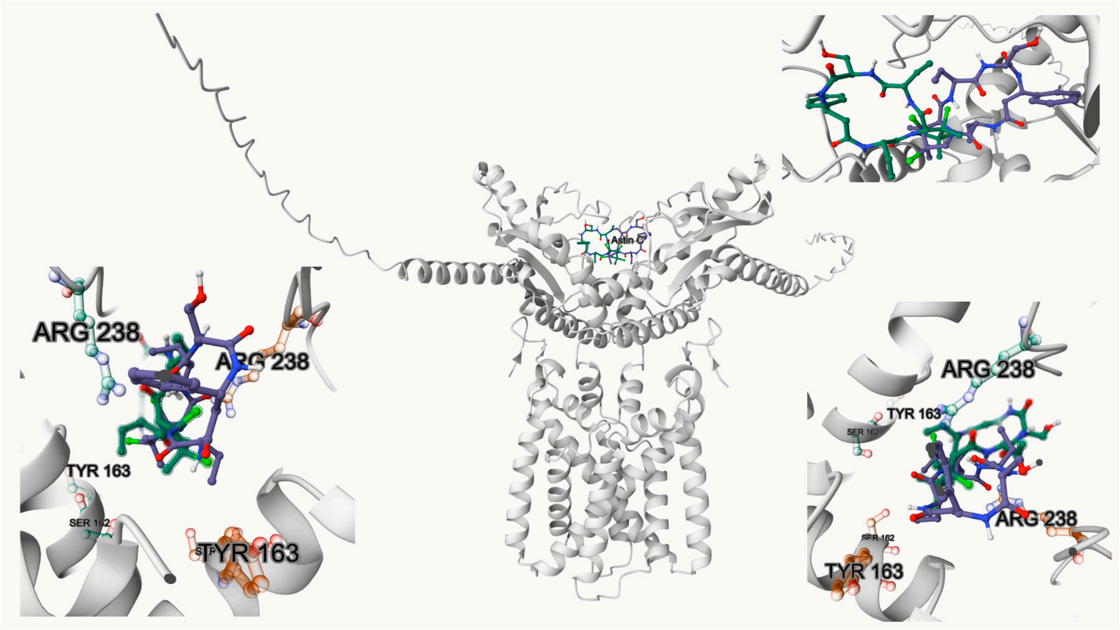

・Figure S2B. Two top-ranked poses of Astin C at the CDN rim (labels: three-letter residue code plus atom index).

Figure S3. Corridor contact-frequency profiles for ExcB across three independent 100 ns replicas (80–100 ns window).

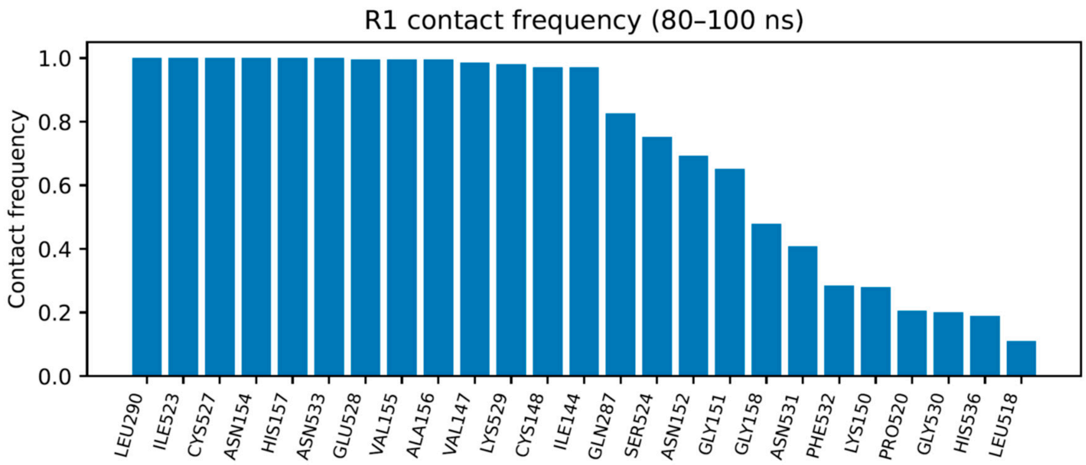

• Figure S3A. Replicate r1 contact-frequency bar plot (80–100 ns).

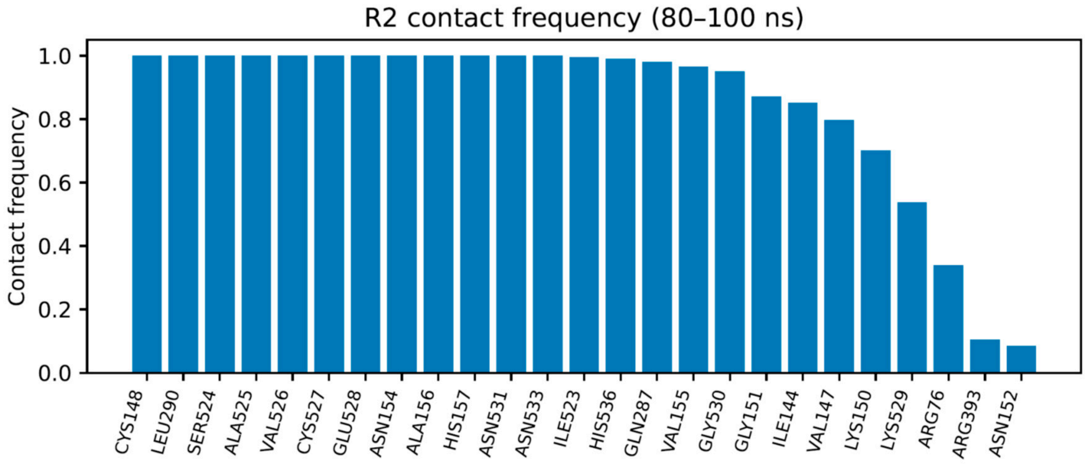

• Figure S3B. Replicate r2 contact-frequency bar plot (80–100 ns).

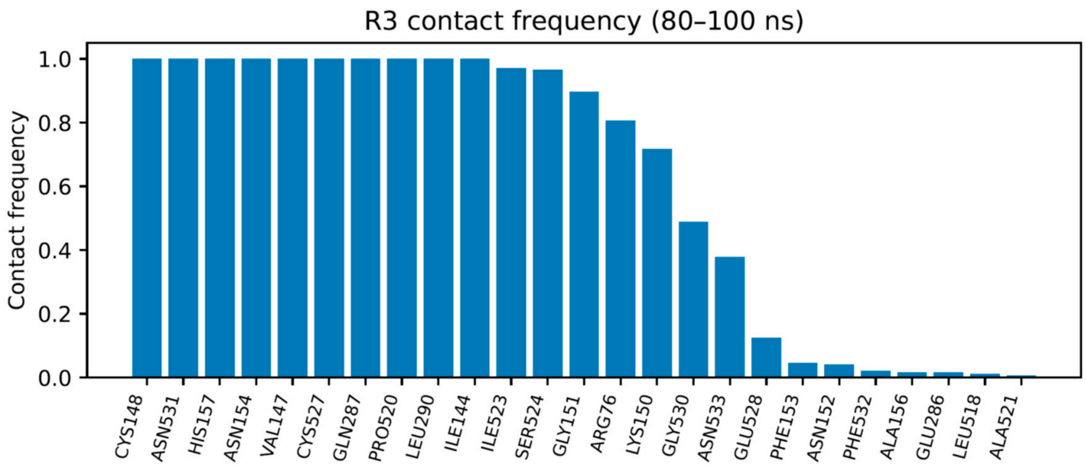

- Figure S3C. Replicate r3 contact-frequency bar plot (80–100 ns).

Bars report per-residue contact frequency between ExcB and STING over the specified time window; plots are shown separately per replicate to visualize reproducibility.

Figure S4. Distance- and hydrogen-bond–based diagnostics for ExcB corridor sampling across three independent 100 ns replicas.

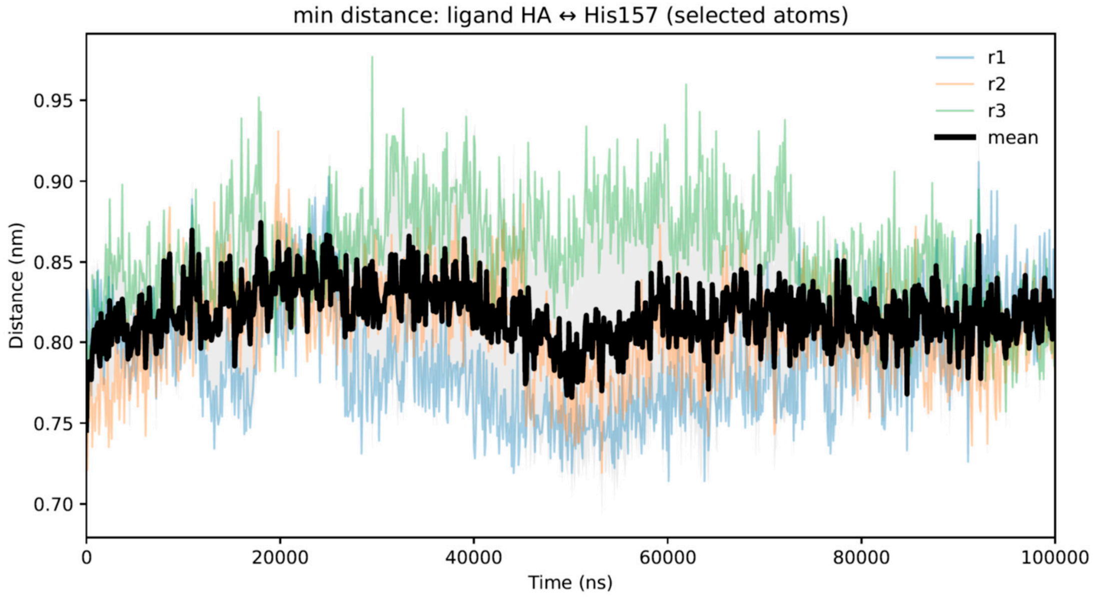

(A) Minimum distance between selected ligand heavy atoms and His157.

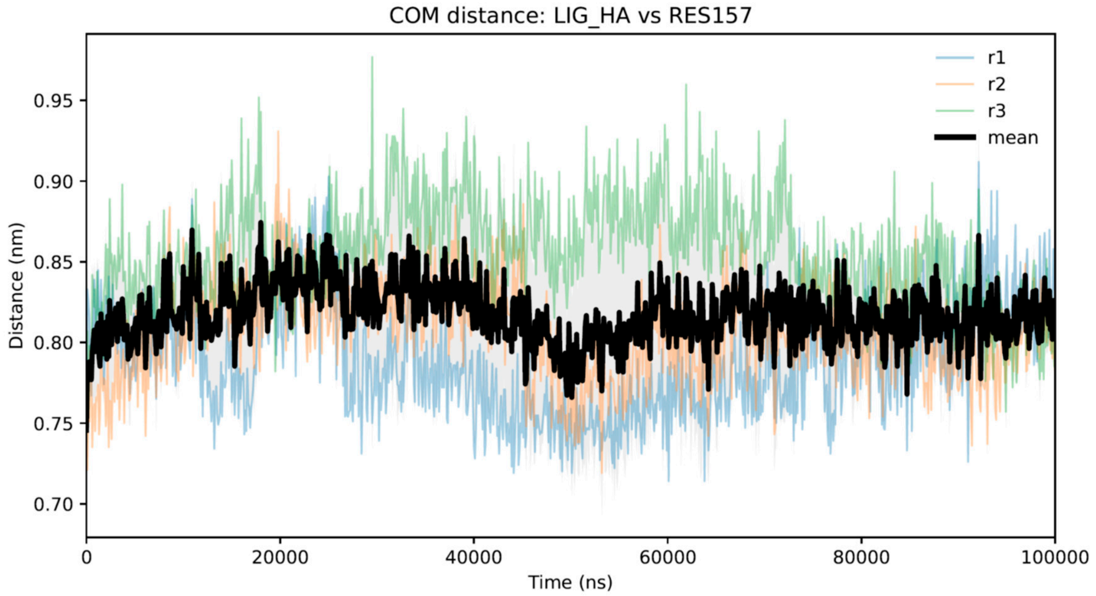

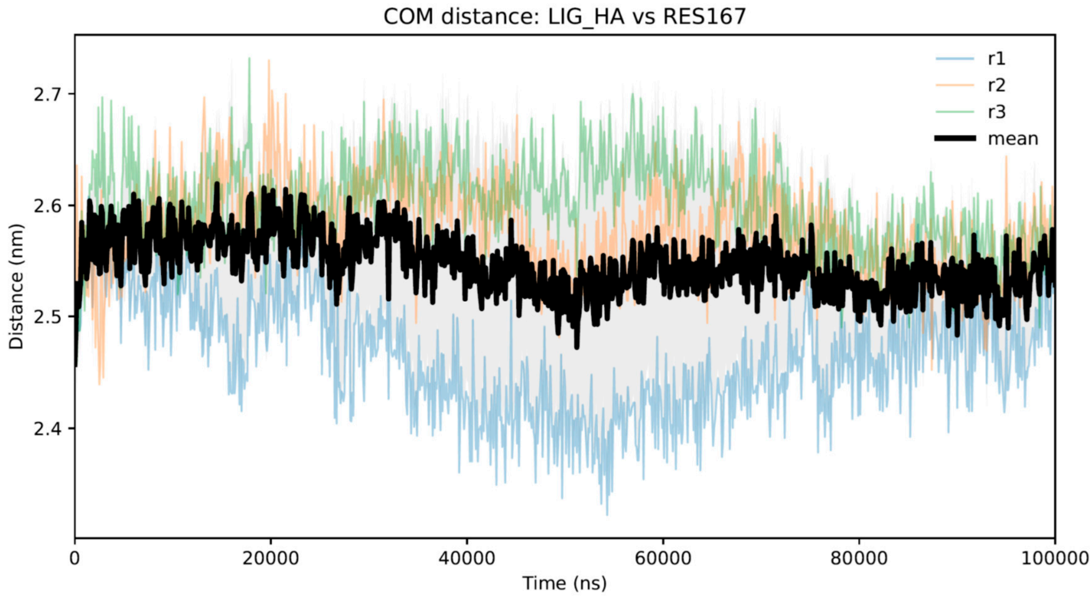

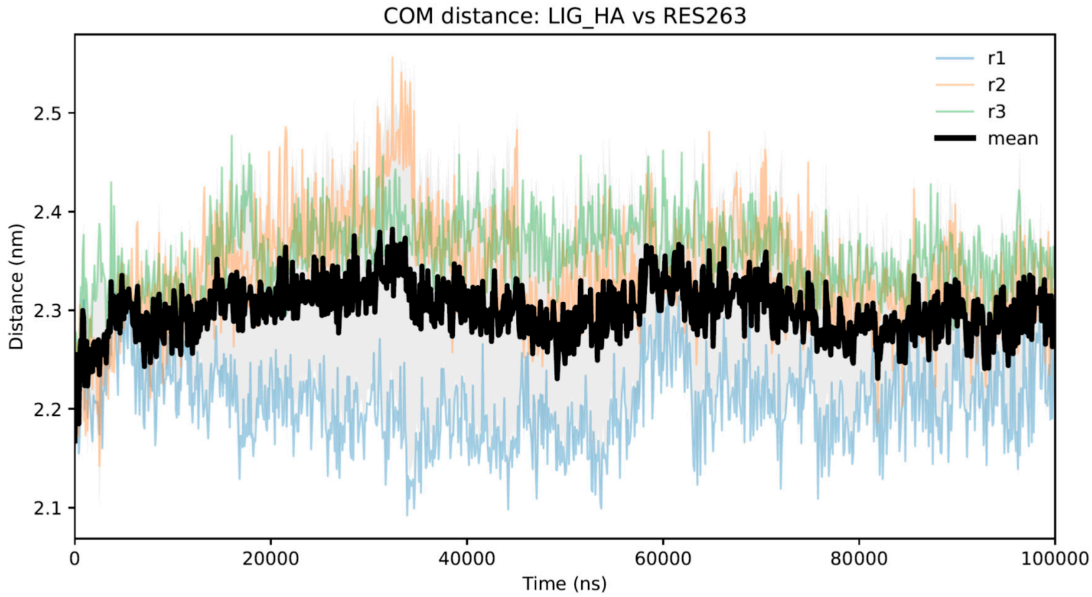

(B–D) Center-of-mass (COM) distance between ligand heavy atoms and reference residues His157, Tyr167, and Thr263, respectively.

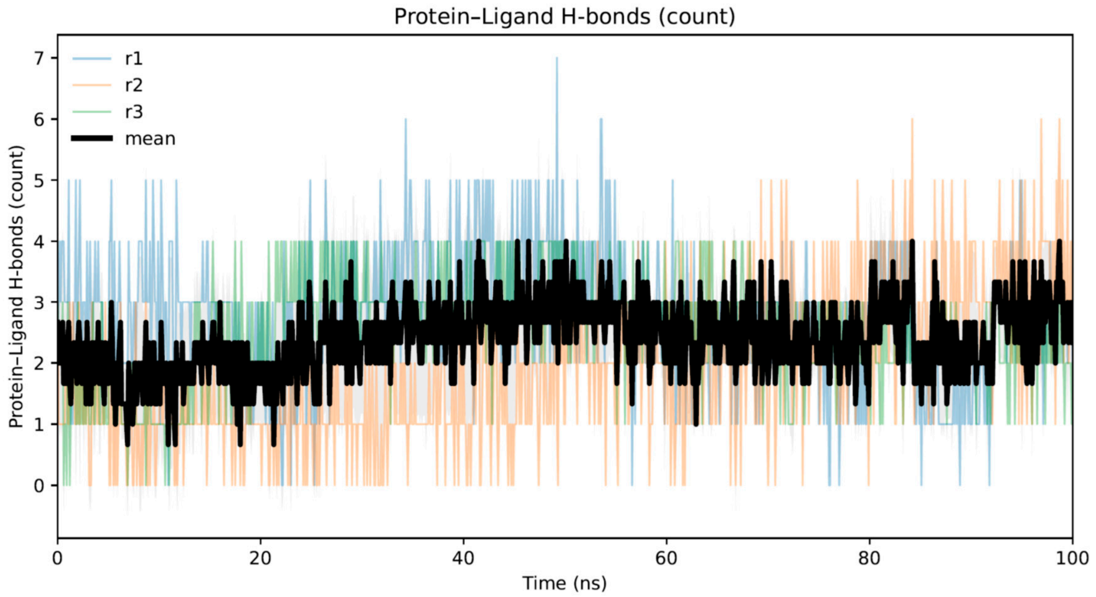

(E) Protein–ligand hydrogen-bond counts over time. Curves show r1–r3 and the across-replicate mean.

Figure S5. Full mdpocket descriptor table for all snapshots across R1–R3 (2 ns sampling).

This table contains the complete mdpocket descriptor output for each analyzed snapshot (1–51 per replica), including pocket volume (pock_volume), solvent-accessible surface area (pock_asa), polar/apolar ASA partitions, convex hull volume, and residue-type counts contributing to the detected pocket. Rows are concatenated across replicas (R1–R3) to enable downstream plotting and summary statistics (e.g., Table S8 and Figure S5). The descriptor definitions follow mdpocket/fpocket conventions; no rescaling or post hoc normalization was applied beyond concatenation and consistent snapshot indexing.

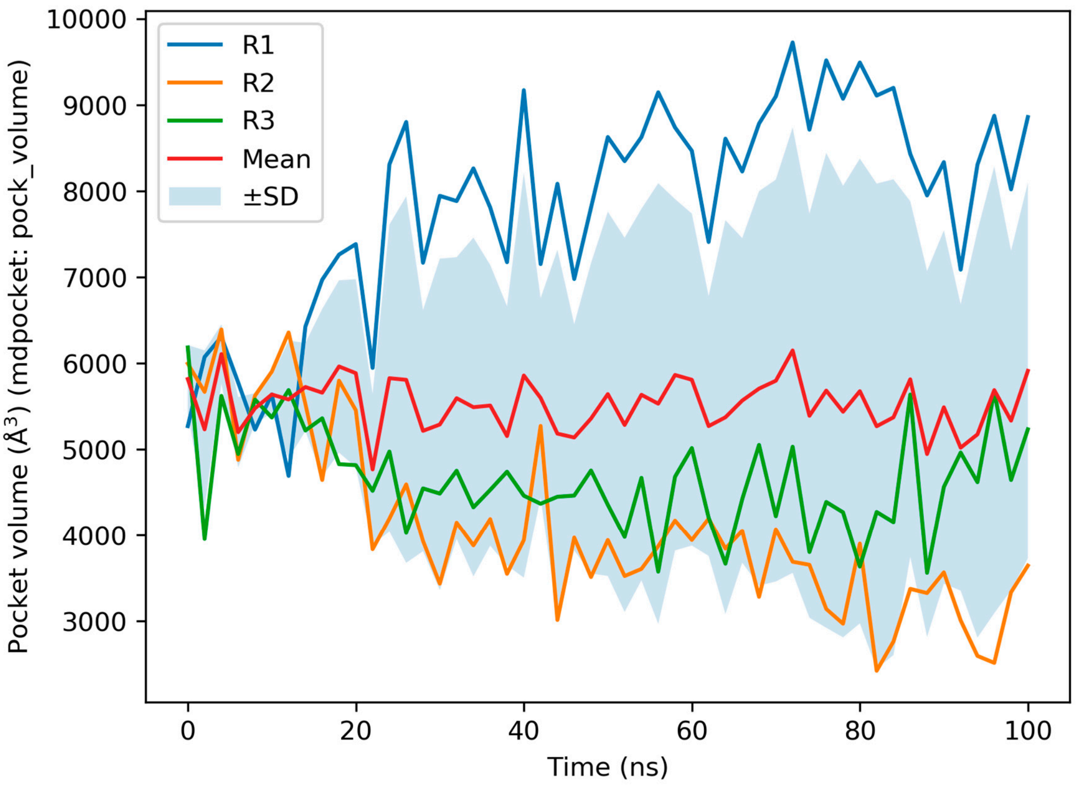

Supplement: Supplementary file 1 [file ijms-27-02243-s001.zip › ijms-4146221-supplementary.pdf]
